# A parametric study on the analysis of thermosolutal convection for magneto-hydrodynamics dependent viscous fluid

**DOI:** 10.1038/s41598-023-42734-6

**Published:** 2023-10-19

**Authors:** Sadia Bano, M. Kamran Alam, Aamir Khan, Abdul Baseer Saqib

**Affiliations:** 1 Department of Mathematics and Statistics, The University of Haripur, Haripur, Pakistan; 2 Department of Mathematics, Nangrahar University, Jalalabad, Nangrahar Afghanistan

**Keywords:** Mechanical engineering, Applied mathematics, Computational science, Software

## Abstract

This article explores the influence of Joule heating and viscous dissipation on the unsteady three-dimensional squeezing flow of Newtonian fluid. The flow in a rotating channel with a lower stretched permeable wall is observed under the influence of a uniform magnetic field. The impact of thermal radiation is also considered. The effects of mass and heat transfer on the squeezing flow of Newtonian fluids are observed and modelled using the four fundamental governing equations of fluid flow: the mass equation, momentum equation, concentration equation, and energy equation. Using the appropriate similarity transformations, the resultant non-linear partial differential equations are then transformed into ordinary differential equations. The analytical strategy is developed using the homotopy analysis method to obtain the series solution. The influence of several physical parameters, including the squeezing parameter, the suction parameter, the magnetic number, the rotation parameter, the Eckert number, the Prandtl number, the Dufour number, the Soret number, the radiation parameter, and the Schmidt number, on the velocity profile, energy, and concentration are also discussed through graphs. Additionally, it is observed that enhancing the top plate’s squeezing impact causes a rise in the velocity profile while lowering the temperature and concentration distribution. It is also found that for the velocity field, increasing the magnetic number shows a decrease in the value of the velocity field along the y- and z-axis, whereas the velocity field along the x-axis exhibits dual behavior, such that it initially falls as the magnetic number intensifies but starts to rise in the upper region of the channel. The impact of the Dufour, Soret, and Eckert numbers on temperature and concentration distribution is also studied. It is found that while these numbers directly affect the temperature distribution, the mass distribution follows the opposite trend. Also, it is noticed that the thermal radiation parameter is an increasing function of temperature and mass distribution. Further, graphs and tables are presented to illustrate an error analysis.

## Introduction

When a substance is pressed out radially between two parallel plates, the flow is known as a squeeze flow. Due to its crucial significance in numerous domains, including biomechanics, artery flow, food processing, mechanical, industrial, and chemical engineering, squeezing flow has drawn substantial interest from many researchers and scientists. The design of lubrication systems, including oil and grease systems, injection and compression shaping, machine tools, automobile engines, pistons, bearings, gears, rolling components ,paper manufacturing, etc., all exhibit this phenomenon. When vertical velocities or normal stresses are imposed because of changing boundaries, these sorts of flows are produced in numerous hydro dynamical devices and equipment. After the seminal study of, several investigations were undertaken in this area. Considering the lubrication approach into account, he speculated about the squeezed flow of a Newtonian fluid. The pioneering work of Stefan was theoretically extended by to power law liquid flowing between squeezed parallel disks. Developed an asymptotic solution for the motion of two rigid spheres squeezing against one another while submerged in an incompressible power-law fluid. He looked at linkages between the two-sphere squeeze problem and the two circular plate squeeze problem.

The term “Magneto-Hydro Dynamics (MHD)” refers to the interaction of conducting fluids with electromagnetic fields. Swedish physicist Alfven^1^ introduced the MHD fluid first. MHD fluid flows have a significant impact on several technical, biological, and industrial processes. It may be utilized for a broad spectrum of applications, including power generators, accelerators, droplet filters, heat exchanger design, electrostatic filters, cooling for reactors, pumps, etc. Additionally, using an MHD fluid as a lubricant is fascinating because it eliminates the unpredictable fluctuation in lubricant viscosity with temperature under specific severe working circumstances. Thus, MHD flows have sparked the attention of many researchers because of their various practical uses. Such as the similarity solution of MHD fluid flow between two squeezed rotating discs studied by Hamza^2^. Siddique et al.^3^ analyzed the unsteady 2-dimensional squeezing flow of viscous MHD fluid between two parallel plates. They used the homotopy perturbation method to obtain the solution. MHD impacts on squeezing flow between parallel discs were studied by Domairry and Aziz^4^. The dimensionless momentum equation’s series solution has been derived using the homotopy perturbation approach. Mohyud-Din et al.^5^ examined the squeeze flow between parallel discs for MHD viscous fluid where one disc is porous and the other is impenetrable. They employed both analytical and numerical techniques to determine the solution. Between two parallel disks with one disk maintained porous, Joneidi et al.^6^ investigated the effect of mass transfer on the flow in the magnetohydrodynamic squeeze film. Hayat et al.^7^ found heat transmission in the squeezing flow between parallel disks when viscous dissipation and MHD effects were present.

Numerous industrial, technical, and geophysical fluid dynamics processes use the flow via a revolving channel. For instance, the measurement of mass flow rate in rotating machinery and devices like radial pumps or compressor compellers depends greatly on the Coriolis force. In addition, an issue of significant interest that has been studied by numerous researchers is the rotating flow of an electrically conducting fluid expressed with a magnetic field. The combined effects of the magnetic field, Hall currents, and free stream velocity on the non-similar boundary layer flow over a moving surface in a rotating fluid were investigated by Takhar et al.^8^. Theoretically, steady boundary layers that grow over the top and bottom surfaces of a revolving channel were addressed Kurosaka et al.^9^.

The flow of incompressible viscous fluids through stretching sheets has drawn a lot of attention from researchers due to its many applications in manufacturing industries and technological processes, including wire drawing, the production of paper and glass fibres, the processing of metal and polymeric materials, etc. Theoretically, Crane^10^ initiated the early research on stretching surfaces as he examined the two-dimensional flow of viscous fluid over a stretching wall. In order to expand the study, Wang^11^ took into account three-dimensional flow caused by stretching a sheet in two orthogonal directions. A great deal of study has been done on the flow over stretched sheets as a result of these ground-breaking hypotheses. To find the precise similarity solutions in this regard, Pavlov^12^ analyzed the MHD flow across a stretched surface while accounting for a uniform magnetic field. Moreover, Munawar et al.^13^ analyzed the unsteady three-dimensional MHD rotational flow compressed between two parallel plates with a bottom stretchy plate kept permeable. They employed the shooting method, a numerical technique, to determine the solution. In magnetohydrodynamics (MHD), Hayat et al.^14^ investigated the unsteady squeezed flow of coupled stress nanostructured materials between two parallel walls. It was shown that larger values of the squeezing parameter result in higher velocity fields, but the temperature and concentration fields show the reverse tendency. The steady state stagnation point flow for a viscous fluid over a contracting or expanding surface with the bottom of the surface heated by convection from a hot fluid was taken into consideration Bachok et al.^15^.

Thermal radiation is the name for the electromagnetic waves that heat-producing substances release. It is regarded as one of the basic processes of heat transmission. Due to its use in physics, engineering, and industrial fields like glass production, furnace design, polymer processing, and gas-cooled nuclear reactors, as well as in space technology like aerodynamic rockets, missiles, propulsion systems, power plants for interplanetary flights, and highly heated spacecraft, the radiation effect in the boundary layer flow is very significant. Thus, in such processes, the effects of heat radiation cannot be disregarded. So the study of heat transfer and thermal radiation in the flow field attracted a lot of researchers’ attention. Hayat et al.^16^ studied the transient two-dimensional squeezing flow of water-based CNTs between two parallel plates. They considered radiative heat flux and the Darcy-Forchheimer porous medium. Sheikholeslami et al.^17^ examined the impact of thermal radiation on the flow of a nanofluid in magnetohydrodynamics between two spinning horizontal plates. The model of nanofluid included the crucial effects of Brownian motion and thermophoresis. The fourth-order Runge-Kutta technique was used to obtain a solution numerically. The two-dimensional magnetohydrodynamic flow of thixotropic fluid toward a stretched surface with varying thermal conductivity and thermal radiation effects was investigated by Hayat et al.^18^. In an incompressible nanofluid flowing across a rotating porous disc with an MHD effect, Rashidi et al.^19^ investigated the entropy generation analysis of flow and heat transmission. Recently, Fiza et al.^20^ reported the Jaffery fluid flow in a three-dimensional rotating channel between two parallel plates. Hall current and MHD effects are also considered. In addition, Alam et al.^21^ investigated the impact of Dufour and Soret on MHD viscous fluid flow between compressing plates when subjected to a variable magnetic field. Unsteady 3D flow was seen in a channel that was spinning. They noticed that the Dufour number had a diminishing influence on the distribution of both concentration and temperature. While the Soret number has a direct impact on concentration, its impact on the distribution of temperature is inverse.

When an electric current travels over a resistance, its energy is transformed into heat, which is known as Joule heating (also known as ohmic dissipation) in electromagnetic fluid dynamics. Moreover, the product of the Hartmann number and the Eckert number, known as the Joule heating effect, has a substantial influence on a variety of industrial applications, including geophysical streams, the petroleum sector, and nuclear engineering. Through the use of electric current movement, Joule heating improves the heat transfer process by diminishing dynamic viscosity and boosting electrical conductivity.The effects of Joule heating on momentum, heat, and mass diffusion characteristics have been widely explored in recent years in hydromagnetic transport phenomena. The impacts of hydromagnetics on the Williamson fluid boundary layer flow across an unsteady permeable stretching sheet were investigated by Hayat et al.^22^. They considered heat radiation and ohmic dissipation, as well as the effects of electric and magnetic fields. The MHD mixed convection flow of thixotropic fluid across a stretched surface was examined by Hayat et al.^18^. Analysis is done on the effects of thermal radiation, thermopheresis, and Joule heating. Using the homotopy analysis approach, series solutions for velocity, temperature, and concentration are obtained. Recently, entropy generation for the flow of water and a combination of water-ethylene glycol as a base fluid and Fe3O4 as a nanoparticle between two spinning stretchable discs with porous media was studied by Hosseinzadeh et al.^23^. They also took into account the impact of MHD, thermal radiation, and Joule heating. Memon et al.^24^ concentrated on the analytical model of the slow squeeze flow of the moderately viscoelastic fluid layer between two circular discs where the upper disc is moving with constant velocity and the lower disc is stationary. The presented problem was resolved by employing the Langlois recursive technique.

Shamshuddin et al.^25^ explored two-dimensional hydromagnetic squeezing flows, heat, and mass transfer via Joule heating and viscous dissipation impacts between two riga plates. The Cattaneo-Christov non-Fourier heat flow model is used since thermal relaxation exists. Additionally, they took into account radiative heat flux and used the variational parameter approach to tackle the problem analytically. Furthermore, Mahanthesh et al.^26^ investigated the impact of viscous dissipation and Joule heating on the three-dimensional flow and heat transfer of a nanofluid across a nonlinear stretching sheet. An elastic sheet was stretched in two lateral directions, causing the fluid which is considered to be electrically conducting to flow. The study came to the conclusion that increasing the radiation parameter causes an increase in the heat transfer rate and that the interaction between thermal radiation and nanoparticle volume fraction stabilizes the development of the thermal boundary layer. The velocity and temperature distributions of a magneto-hydrodynamic flow squeezed between two parallel discs with suction or injection were examined by Parand et al.^27^. They used polynomials from the shifted Chebyshev, Euler, and Bessel families to address the problem using the collocation approach. The incompressible axisymmetric squeezing flow of second-grade fluid between two porous disks was studied by Hayat et al.^28^. While conducting a heat transfer study, thermal radiation and convective boundary conditions are taken into account. They reported that the temperature profile increases as the radiation parameter increases. Ahmed et al.^29^ analyzed the Corban nanotube-containing nanofluid that is squeezed between Riga plates. The effects of heat radiation and viscous dissipation are also taken into consideration. The Adomian decomposition approach and the Runge-Kutta method were employed, respectively, for the model’s analytical and numerical treatment. The effects of an induced magnetic field on the peristaltic flow of an electrically conducting Jeffrey fluid in an asymmetric channel with a partial slip condition have been explored Nadeem and Akram^30^.

The impact of heat and mass transfer on the two-dimensional steady flow of an MHD Oldroyd-B fluid through a stretched surface has been studied by Hayat et al.^31^. Thermal radiation, Joule heating, and thermophoresis are also accounted for. Analytical investigation of Soret and Dufour effects in the MHD three-dimensional boundary layer flow of second-grade fluid carried on by an exponentially extending surface with thermal radiation Hayat et al.^32^. The three-dimensional flow of the nanofluid model with microorganisms across a spinning disc with power-law stretching analyzed by Chen et al.^33^. It has been discovered that the power-law stretching index has a significant impact on the flow as well as the heat and mass transfer. Later on, Muzara and Shateyi^34^ examined the laminar boundary layer flow of a MHD Jeffrey fluid flowing over a vertical permeable plate. A heat source or sink, as well as viscous dissipation both affect flow. Shamim et al.^35^ scrutinized the power-law fluid flow across the horizontal stretched cylinder. In heat transfer, the combined effects of constant thermal conductivity and viscous dissipation are examined. They noticed that the thickness of the thermal boundary layer increased as the Eckert number increased.The Hall current was taken into consideration by Krishna et al.^36^ when modelling the hydromagnetic squeezing flow of a water-based nanofluid through a porous channel between two parallel disks.

Considering the literature mentioned above and some new (Fehmi Gamaoun, et. al., 2023, & M. Veera Krishna et. al., 2021), the current study is designed to scrutinise the three-dimensional squeezed flow of Newtonian fluid in a rotating channel with a lower stretchy permeable wall subject to Joule heating and viscous dissipation effects. Thermal radiation is also included. The effects of the magnetic field and Coriolis force are still reflected in the momentum equation. This research builds on the work of Munawar et al.^13^ by incorporating salient features of heat and mass transfer. In addition, to develop an analytical approach to a non-linear flow problem, the homtopy analysis method is applied using the mathematica package BVPh 2.0 Zhao et al.^37^. From the literature ^38–46^ it is confidently claim that no work has been done on the present work. The present research aims to offer a comprehensive solution to these specific problems and introduce a new approach to the analysis of flow between parallel plates, which has not been explored before according to the author’s knowledge. This work seeks to contribute valuable insights to the field and serve as a source of motivation for further research endeavors in this area.

## Formulation of the problem

**Figure 1:**
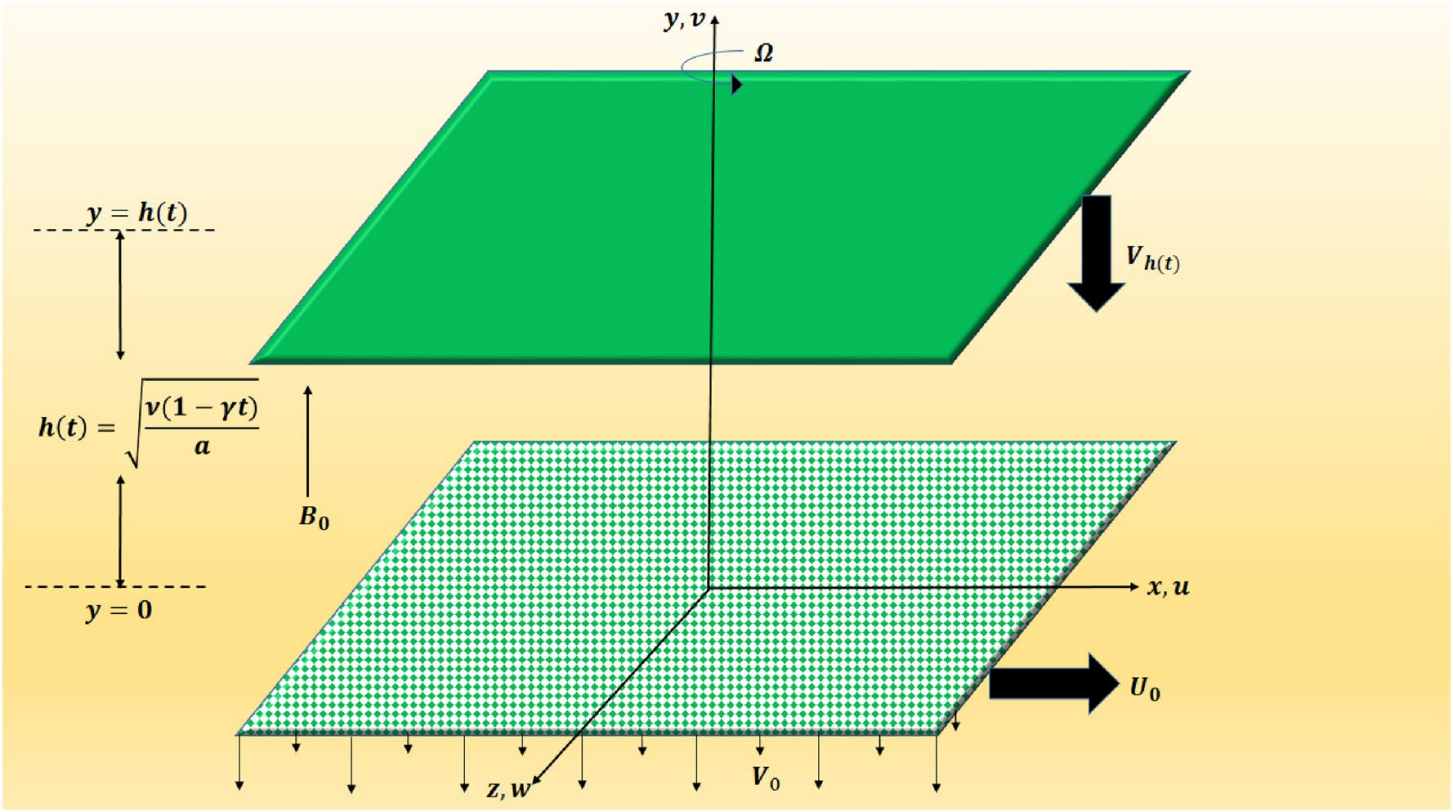
Geometry of the problem.

We assume unsteady three dimensional rotating laminar flow of an in-compressible Newtonian fluid between two squeezing plates apart by a distance of $$ h(t)=\sqrt{\dfrac{\nu \left( 1-\gamma t\right) }{a}} $$. The top plate at $$ y=h(t) $$ is approaching with velocity $$ V_h=\dfrac{dh}{dt}=-\dfrac{\gamma }{2}\sqrt{\dfrac{\nu }{a\left( 1-\gamma t\right) }} $$ towards as well as away from the porous plate at bottom, placed at $$ y=0 $$. The bottom plate has the ability to stretch in the $$ x- $$direction with velocity $$ U_{0}=\dfrac{ax}{1-\gamma t} $$. Additionally, the top plate spins around the $$ y- $$axis with an angular velocity $$ \Omega =\dfrac{\omega j}{1-\gamma t} $$, while the bottom plate suctions the fluid with a velocity $$\dfrac{ -V_{0}}{1-\gamma t} $$. In the $$ y-$$direction, an uniform magnetic field of intensity $$ B\left( t\right) =\dfrac{B_{0}}{\sqrt{1-\gamma t}}$$ is likewise applied. The physical structure of the flow problem is depicted in Fig. 1. We use a Cartesian coordinate system where $$y-$$axis is normal to both plates, $$ xz- $$plane is aligned with the bottom plate and origin is set at the middle of the bottom plate. The fluid motion is caused by the $$ x-$$direction stretching, the top plate rotating, and the squeezing impact. The heat and mass transport properties are considered in the presence of Soret and Dufour effects. Both Joule heating and viscous dissipation influences on fluid flow are analyzed. Further, the thermal radiation effect is also preserved. The induced magnetic and electric fields are supposed to be omitted on the basis of the low Reynolds number. The gravitational impact is likewise minimal. The equations of motion incorporate the effects of the Lorentz and Coriolis forces. Accordingly, the component form of the equations for continuity, momentum, energy, and concentration is defined by: Munawar et al.^13^ and Alam et al.^47^

Conservation of Mass Equation (Papanastasiou, Tasos, Georgios Georgiou, Viscous fluid flow. CRC press, 2021, White^48^):

1 $$\begin{aligned} \dfrac{\partial u}{\partial x}+\dfrac{\partial v}{\partial y}+\dfrac{\partial w}{\partial z}=0 \end{aligned}$$

X-Component of the Conservation of Momentum Equation:

2 $$\begin{aligned} \rho \left[ \dfrac{\partial u }{\partial t} + u\dfrac{\partial u }{ \partial x} + v\dfrac{\partial u }{\partial y } + w \dfrac{\partial u }{\partial z}+\dfrac{2\omega }{1-\gamma t}w \right] =-\dfrac{\partial P }{\partial x} +\mu \left[ \dfrac{\partial ^2 u }{\partial x^2} +\dfrac{\partial ^2 u }{\partial y^2 }+\dfrac{\partial ^2 u }{\partial z^2} \right] -\dfrac{\sigma B_0^{2}}{1-\gamma t}u \end{aligned}$$

Y-Component of the Conservation of Momentum Equation:

3 $$\begin{aligned} \rho \left[ \dfrac{\partial v }{\partial t}+u\dfrac{\partial v }{\partial x}+v\dfrac{\partial v }{ \partial y }+w\dfrac{\partial v }{\partial z} \right] =-\dfrac{\partial P }{\partial y } +\mu \left[ \dfrac{\partial ^2 v }{\partial x^2} + \dfrac{\partial ^2 v }{\partial y^2 }+\dfrac{\partial ^2 v }{\partial z^2} \right] \end{aligned}$$

Z-Component of the Conservation of Momentum Equation:

4 $$\begin{aligned} \rho \left[ \dfrac{\partial w }{\partial t}+u\dfrac{\partial w }{\partial x}+v\dfrac{\partial w }{ \partial y }+w\dfrac{\partial w }{\partial z}-\dfrac{2\omega }{1-\gamma t}u \right] =-\dfrac{\partial P }{\partial z } +\mu \left[ \dfrac{\partial ^2 w }{\partial x^2} +\dfrac{\partial ^2 w }{\partial y^2 }+\dfrac{\partial ^2 w }{\partial z^2} \right] -\dfrac{\sigma B_0^{2}}{1-\gamma t}w \end{aligned}$$

Heat Transfer Equation:

5 $$\begin{aligned} \begin{array}{c} \rho \left[ \dfrac{\partial T}{\partial t}+u\dfrac{\partial T}{\partial x}+v\dfrac{\partial T}{\partial y}+w\dfrac{\partial T}{\partial z} \right] =\dfrac{\kappa }{C_p}\left[ \dfrac{\partial ^2 T}{\partial x^2}+\dfrac{\partial ^2 T}{\partial y^2}+\dfrac{\partial ^2 T}{\partial z^2}\right] - \dfrac{1}{C_p}\nabla q_r+\dfrac{D\kappa _t}{C_sC_p}\left[ \dfrac{\partial ^2 C}{\partial x^2}+\dfrac{\partial ^2 C}{\partial y^2}+\dfrac{\partial ^2 C}{\partial z^2} \right] +\\ \dfrac{\mu }{C_p}\left[ 2\left( \left( \dfrac{\partial u}{\partial x}\right) ^{2}+\left( \dfrac{\partial v}{\partial y}\right) ^{2}+\left( \dfrac{\partial w}{\partial z}\right) ^{2}\right) +\left( \dfrac{\partial u}{\partial y}+\dfrac{\partial v}{\partial x}\right) ^{2}+ \left( \dfrac{\partial u}{\partial z}+\dfrac{\partial w}{\partial x}\right) ^{2}+\left( \dfrac{\partial v}{\partial z}+\dfrac{\partial w}{\partial y}\right) ^{2}\right] +\\ \dfrac{\sigma B_0^{2}}{C_p(1-\gamma t)}(u^{2}+w^{2}) \end{array} \end{aligned}$$

Mass Transfer Equation:

6 $$\begin{aligned} \rho \left[ \dfrac{\partial C}{\partial t}+u\dfrac{\partial C}{\partial x}+v\dfrac{\partial C}{\partial y}+w\dfrac{\partial C}{\partial z} \right] =D\left[ \dfrac{\partial ^2 C}{\partial x^2}+\dfrac{\partial ^2 C}{\partial y^2}+\dfrac{\partial ^2 C}{\partial z^2}\right] + \dfrac{D\kappa _t}{C_sT_m}\left[ \dfrac{\partial ^2 T}{\partial x^2}+\dfrac{\partial ^2 T}{\partial y^2}+\dfrac{\partial ^2 T}{\partial z^2} \right] \end{aligned}$$

## Boundary conditions

The boundary conditions are chosen as: Munawar et al.^13^ and Hayat et al.^14^

7 $$ \begin{aligned}{}&u=U_0=\dfrac{ax}{1-\gamma t}, \hspace{0.2in}v=-\dfrac{V_0}{1-\gamma t},\hspace{0.2in} w=0, \hspace{0.2in} T=T_{0}, \hspace{0.2in}C=C_{0} \hspace{0.2in} {\text {at}}\hspace{0.2in} y=0\\&u=0,\hspace{0.2in}v=V_h=\dfrac{dh(t)}{dt}, \hspace{0.2in} w=0, \hspace{0.2in} T=T_0+\dfrac{T_0}{1-\gamma t}, \hspace{0.2in} C=C_0+\dfrac{C_0}{1-\gamma t}, \hspace{0.2in}{\text {at}}\hspace{0.2in} y = h\left( t\right) \\ {}&{\text {where}}\hspace{0.2in} h(t)=\sqrt{\dfrac{\nu \left( 1-\gamma t\right) }{a}}. \end{aligned}$$

where *u*, *v*, and *w* are velocity components along the *x*, *y* and $$ z-$$directions, respectively. *a*, $$ \gamma $$, $$ V_0>0 $$, $$ T_0 $$, and $$ C_0 $$, respectively stand for the stretching rate of the bottom plate, squeezing rate of top plate, suction, temperature, and concentration at bottom plate, while $$ \rho $$, $$ \mu $$, *P*, and $$ \sigma $$ denote density of fluid, dynamic viscosity, pressure, and electrical conductivity respectively, *T* is temperature, *C* is concentration, *D* is mass diffusion coefficient, $$\kappa $$ is thermal conductivity, $$ \kappa _t $$ is thermal diffusion, $$ C_p $$ is specific heat, $$ C_s $$ is concentration sensitivity, $$ T_m $$ is fluid mean temperature and $$ q_r $$ is the radiative heat flux in $$ y- $$direction, so that $$ \nabla q_r=\dfrac{\partial q_r}{\partial y} $$.

Utilizing the Rosseland approximation, the radiative heat flux $q_r$ may be written as, Hayat et al.^31^

8 $$\begin{aligned} q_r = -\dfrac{4\sigma ^{s}}{\kappa ^{a}}\dfrac{\partial T^{4}}{\partial y}, \end{aligned}$$

where $\sigma ^{s}$ stand for Stefan-Boltzman constants and $\kappa ^{a}$ for the Rosseland mean absorption coefficient. Since there are no significant temperature variations between the fluid phases, $T^{4}$ may be expressed as a linear function of temperature. As a result, expanding $T^{4}$ in a Taylor series around $T_{0}$ and excluding higher order terms greater than the first degree in $(T-T_{0})$, we get

9 $$\begin{aligned} T^{4} \cong 4T_{0}^3T-3T_{0}^4 \end{aligned}$$

From Eq. (8), the partial derivative of $q_r$ with respect to *y* is,

10 $$\begin{aligned} \dfrac{\partial q_r}{\partial y} = -\dfrac{4\sigma ^{s}}{3\kappa ^{a}} \dfrac{\partial ^{2}T^{4}}{\partial y^{2}} \end{aligned}$$

By substituting Eq. (9) into Eq. (10) we obtain,

11 $$\begin{aligned}{} & {} \dfrac{\partial q_r}{\partial y} = -\dfrac{16\sigma ^{s}T_{0}^3}{3\kappa ^{a}} \dfrac{\partial ^{2}T}{\partial y^{2}} \end{aligned}$$

12 $$\begin{aligned}{} & {} \Rightarrow \nabla q_r=-\dfrac{4\sigma ^{s}}{3\kappa ^{a}} \dfrac{\partial ^{2}T^{4}}{\partial y^{2}},\,\,\,as\,\,\,\dfrac{\partial q_r}{\partial y}= \nabla q_r. \end{aligned}$$

The above set of partial differential equations are difficult to solve using numerical/analytical methods. Therefore, these partial differential equations $(1-6)$ can be converted into ordinary differential equations by applying the similarity transformations shown below, Munawar et al.^13^ and Hayat et al.^14^

13 $$ \begin{aligned}{}&u=U_0f'(\eta ), \hspace{0.2in}v=-\sqrt{\dfrac{a\nu }{1-\gamma t}}f(\eta ),\hspace{0.2in} w=U_0g(\eta ), \hspace{0.2in} T=T_0+\dfrac{T_0}{1-\gamma t} \theta (\eta ),\hspace{0.2in}C=C_0+\dfrac{C_0}{1-\gamma t} \phi (\eta ),\\ {}&\eta =\frac{y}{h(t)},\hspace{0.2in} {\text {where}}\hspace{0.1in}\eta \hspace{0.1in}{\text {is the non-dimensional similarity variable.}} \end{aligned}$$

The continuity Eq. (1) is identically verified after normalization, and the pressure terms are removed from the resulting momentum equations via cross-differentiation. Finally, the Eqs. (2–7) have been transformed to following coupled system of ordinary differential equations.

14 $$\begin{aligned}{} & {} f^{''''}+ff^{'''}-ff^{''}-\dfrac{S_q}{2}\left( 3f^{''}+\eta f^{'''}\right) -2\Omega g^{'}-M^{2}f^{''}=0 \end{aligned}$$

15 $$\begin{aligned}{} & {} g^{''}+fg^{'}-f^{'}g-\dfrac{S_q}{2}\left( 2g+ \eta g^{'} \right) +2 \Omega f^{'}-M^{2}g =0 \end{aligned}$$

16 $$ \begin{aligned}{}&\left( 3+4R_d\right) \theta ^{''}+ 3P_r \left( D_u \phi ^{''}+ \theta ^{'}f-\dfrac{S_q}{2}\left( \eta \theta ^{'}+2 \theta \right) \right) +\\ {}&3P_rE_c\left( {f^{''}}^2+{g^{'}}^2+\delta ^{2}\left( 4{f^{'}}^2+g^{2}\right) +M^{2}\left( {f^{'}}^2+g^{2}\right) \right) =0  \end{aligned}$$

17 $$\begin{aligned}{} & {} \phi ^{''}+S_c \left( f\phi ^{'}-\dfrac{S_q}{2}\left( 2\phi +\eta \phi ^{'}\right) +S_r\theta ^{''}\right) =0 \end{aligned}$$

with the accompanying boundary conditions:

18 $$ \begin{aligned}{}&f(0)=S, \hspace{0.3cm} f'(0) = 1,\hspace{0.3cm} g(0) = 0,\hspace{0.3cm} \theta (0) = 0,\hspace{0.3cm} \phi (0) = 0, \\ {}&f(1) = \dfrac{S_q}{2},\hspace{0.3cm} f'(1) = 0,\hspace{0.3cm}g(1)= \Omega ,\hspace{0.3cm} \theta (1) = 1,\hspace{0.3cm} \phi (1) = 1.  \end{aligned}$$

where the derivative with respect to $\eta$ is represented by the prime. $S_q$ stands for squeezing number, *M* for magnetic number (Hartmann number), $\Omega$ for rotation parameter, *S* for suction parameter, $P_r$ is the Prandtl number, $E_c$ represents Eckert number, $D_u$ is Dufour number, $R_d$ is the radiation parameter, $\delta$ is the length parameter, $S_r$ is Soret number, and $S_c$ is Schmidt number.

These parameters are defined by,

19 $$ \begin{aligned}{}&S_q=\dfrac{\gamma }{a}, \hspace{0.2cm} M=\dfrac{\sigma B_0^{2} }{\rho a},\hspace{0.2cm} \Omega =\dfrac{\omega }{a},\hspace{0.2cm} S=\dfrac{V_0}{ah},\hspace{0.2cm} P_r=\dfrac{\nu C_p}{\kappa }=\dfrac{\rho \nu C_p}{\kappa },\hspace{0.2cm} E_c=\dfrac{U_0^{2}\left( 1-\gamma t\right) }{C_p T_0},\hspace{0.2cm} D_u=\dfrac{D{\kappa }_t C_0}{C_s C_p \nu T_0}, \\ {}&R_d= \dfrac{4\sigma ^s {T_u}^{3}}{\kappa ^a \kappa },\hspace{0.2cm} \delta ^2=\dfrac{\nu \left( 1- \gamma t\right) }{ax^{2}},\hspace{0.2cm} S_r=\dfrac{D\kappa _t T_0}{T_m \nu C_0 C_s},\hspace{0.2cm}S_c=\dfrac{\nu }{D}=\dfrac{\rho \nu }{D}.  \end{aligned}$$

## Method of solution

An analytical technique is used to find the solution of the Eqs. (14–18), known as Homotopy Analysis Method. Expressing the functions $f_n$, $g_n$, $\theta _n$ and $\phi _n$ (where *f*, *g*, $\theta$ and $\phi$ are the functions of $\eta$) by a set of base functions ${\eta ^{K}, K \ge 0}$, as

20 $$\begin{aligned}{} & {} f_{n} = \sum _{K=0}^{\infty } a_{K}\eta ^{K} \end{aligned}$$

21 $$\begin{aligned}{} & {} g_{n} = \sum _{K=0}^{\infty } b_{K}\eta ^{K} \end{aligned}$$

22 $$\begin{aligned}{} & {} \theta _{n} = \sum _{K=0}^{\infty } c_{K}\eta ^{K} \end{aligned}$$

23 $$\begin{aligned}{} & {} \phi _{n} = \sum _{K=0}^{\infty } d_{K}\eta ^{K} \end{aligned}$$

where the constant co-efficients $a_{K}$, $b_{K}$, $c_{K}$ and $d_{k}$ are to be determined. Initial approximations are chosen as follows:

24 $$\begin{aligned}{} & {} f_{0} = (-S_q+2*S +1)*\eta ^3 +(-2+\frac{3}{2}*S_q-3*S)*\eta ^2+\eta +S \end{aligned}$$

25 $$\begin{aligned}{} & {} g_{0} = 0 \end{aligned}$$

26 $$\begin{aligned}{} & {} \theta _{0} = \eta \end{aligned}$$

27 $$\begin{aligned}{} & {} \phi _{0} = \eta \end{aligned}$$

the auxiliary operators has the following form,

28 $$ \begin{aligned}{}&{\pounds }_{f} = \frac{\partial ^{4}}{\partial \eta ^{4}},{\hspace{0.3cm}} {\pounds }_{g} = \frac{\partial ^{2}}{\partial \eta ^{2}},{\hspace{0.3cm}} {\pounds }_{\theta } = \frac{\partial ^{2}}{\partial \eta ^{2}}, {\hspace{0.3cm}}{\pounds }_{\phi } = \frac{\partial ^{2}}{\partial \eta ^{2}}  \end{aligned}$$

with the following properties

29 $$\begin{aligned}{} & {} {\pounds }_{f}( k_{1^*}\eta ^3 + K_{2^*}\eta ^2 + K_{3^*}\eta + K_{4^*}) = 0 \end{aligned}$$

30 $$\begin{aligned}{} & {} {\pounds }_{g}( K_{5^*}\eta + K_{6^*}) = 0 \end{aligned}$$

31 $$\begin{aligned}{} & {} {\pounds }_{\theta }( K_{7^*}\eta + K_{8^*}) = 0 \end{aligned}$$

32 $$\begin{aligned} {\pounds }_{\phi }( K_{9^*}\eta + K_{10^*}) = 0 \end{aligned}$$

where $K_{1^*}$ upto $K_{10^*}$ all represents arbitrary constants.

We can obtain the *Zeroth* order deformation as:

33 $$\begin{aligned}{} & {} ( 1 - s ){\pounds }_{f}[f(\eta ; s) - f_{0}(\eta )] = s\hbar _{f}\aleph _{f}[f(\eta ; s), g(\eta ; s)] \end{aligned}$$

34 $$\begin{aligned}{} & {} \begin{array}{c} ( 1 - s ){\pounds }_{g}[g(\eta ; s) - g_{0}(\eta )] = s\hbar _{g}\aleph _{g}[f(\eta ; s), g(\eta ; s)] \end{array} \end{aligned}$$

35 $$\begin{aligned}{} & {} ( 1 - s ){\pounds }_{\theta }[\theta (\eta ; s) - \theta _{0}(\eta )] = s\hbar _{\theta }\aleph _{\theta }[f(\eta ; s), g(\eta ; s), \theta (\eta ; s), \phi (\eta ; s)] \end{aligned}$$

36 $$\begin{aligned}{} & {} \begin{array}{c} ( 1 - s ){\pounds }_{\phi }[\phi (\eta ; s) - \phi _{0}(\eta )] = s\hbar _{\phi }\aleph _{\phi }[f(\eta ; s), \theta (\eta ; s), \phi (\eta ; s)] \end{array} \end{aligned}$$

From Eqs. (14–17),the nonlinear operators are define as,

37 $$\begin{aligned}{} & {} \begin{array}{c} \aleph _{f}[f(\eta ; s), g(\eta ; s)] = \frac{\partial ^{4}f(\eta ; s)}{\partial \eta ^{4}}+f(\eta ; s)\frac{\partial ^{3}f(\eta ; s)}{\partial \eta ^{3}}-f(\eta ; s)\frac{\partial ^{2}f(\eta ; s)}{\partial \eta ^{2}} - \frac{S_q}{2}\bigg (3 \frac{\partial ^{2}f(\eta ; s)}{\partial \eta ^{2}}+\\ \eta \frac{\partial ^{3}f(\eta ; s)}{\partial \eta ^{3}} \bigg )-2 \Omega \frac{\partial g(\eta ; s)}{\partial \eta } -M^{2}\frac{\partial ^{2}f(\eta ; s)}{\partial \eta ^{2}} \end{array} \end{aligned}$$

38 $$\begin{aligned}{} & {} \begin{array}{c} \aleph _{g}[f(\eta ; s), g(\eta ; s)] = \frac{\partial ^{2}g(\eta ; s)}{\partial \eta ^{2}}+f(\eta ; s)\frac{\partial g(\eta ; s)}{\partial \eta }-\frac{\partial f(\eta ; s)}{\partial \eta }g(\eta ; s) -\\ \frac{S_q}{2}\bigg (2g(\eta ; s) + \eta \frac{\partial g(\eta ; s)}{\partial \eta }\bigg )+2 \Omega \frac{\partial f(\eta ; s)}{\partial \eta }-M^{2}g(\eta ; s) \end{array} \end{aligned}$$

39 $$\begin{aligned}{} & {} \begin{array}{c} \aleph _{\theta }[f(\eta ; s), g(\eta ; s), \theta (\eta ; s), \phi (\eta ; s)] = \bigg (3+4R_{d}\bigg )\frac{\partial ^{2}\theta (\eta ; s)}{\partial \eta ^{2}}+3P_{r}\bigg (D_{u}\frac{\partial ^{2}\phi (\eta ; s)}{\partial \eta ^{2}}+\frac{\partial \theta (\eta ; s)}{\partial \eta }f(\eta ; s)-\\ \frac{S_q}{2}\bigg (\eta \frac{\partial \theta (\eta ; s)}{\partial \eta }+2\theta (\eta ; s)\bigg ) \bigg )+3P_{r}E_{c}\bigg (\bigg (\frac{\partial ^{2}f(\eta ; s)}{\partial \eta ^{2}}\bigg )^{2}+\bigg (\frac{\partial g(\eta ; s)}{\partial \eta }\bigg )^{2}+{\delta }^{2}\bigg (4\bigg (\frac{\partial f(\eta ; s)}{\partial \eta }\bigg )^{2}+\left( g(\eta ; s)\right) ^{2}\bigg )+\\ M^{2}\bigg (\bigg (\frac{\partial f(\eta ; s)}{\partial \eta }\bigg )^{2}+\left( g(\eta ; s)\right) ^{2}\bigg ) \bigg ) \end{array} \end{aligned}$$

40 $$\begin{aligned}{} & {} \begin{array}{c} \aleph _{\phi }[f(\eta ; s), \theta (\eta ; s), \phi (\eta ; s)] = \frac{\partial ^{2} \phi (\eta ; s)}{\partial \eta ^{2}}+S_c \bigg (f(\eta ; s)\frac{\partial \phi (\eta ; s)}{\partial \eta }-\frac{S_q}{2}\bigg (2\phi (\eta ; s)+\eta \frac{\partial \phi (\eta ; s)}{\partial \eta }\bigg )+S_r\frac{\partial ^{2} \theta (\eta ; s)}{\partial \eta ^{2}}\bigg ) \end{array} \end{aligned}$$

where *s* is a fix parameter, $\hbar _f$, $\hbar _g$, $\hbar _\theta$ and $\hbar _\phi$ are the nonzero auxiliary parameter, while nonlinear parameters are $\aleph _f$, $\aleph _g$, $\aleph _\theta$ and $\aleph _\phi$.

For $s = 0$ and $s = 1$, we have

41 $$ \begin{aligned}{}&f(\eta ,0) = f_0, \,\,\,\, g(\eta ,0) = g_0, \,\,\,\, \theta (\eta ,0) = \theta _0, \,\,\,\, \phi (\eta ,0) = \phi _0 \\ {}&f(\eta ,1) = f(\eta ), \,\,\,\, g(\eta ,1) = g(\eta ), \,\,\,\, \theta (\eta ,1) = \theta (\eta ), \\ {}&\phi (\eta ,1) = \phi (\eta )  \end{aligned}$$

so as *s* varies from 0 to 1, exact solution $f(\eta )$, $g(\eta )$, $\theta (\eta )$ and $\phi (\eta )$ can be obtained from initial guesses $f_0$, $g_0$, $\theta _0$ and $\phi _0$ to respectively.

For these functions the Taylor’s series are given by:

42 $$\begin{aligned}{} & {} f(\eta ;s) = f_0 + \sum _{n=1}^{\infty }s^nf_n(\eta ) \end{aligned}$$

43 $$\begin{aligned}{} & {} g(\eta ;s) = g_0 + \sum _{n=1}^{\infty }s^ng_n(\eta ) \end{aligned}$$

44 $$\begin{aligned}{} & {} \theta (\eta ;s) = \theta _0 + \sum _{n=1}^{\infty }s^n\theta _n(\eta ) \end{aligned}$$

45 $$\begin{aligned}{} & {} \phi (\eta ;s) = \phi _0 + \sum _{n=1}^{\infty }s^n\phi _n(\eta ) \end{aligned}$$

46 $$\begin{aligned}{} & {} \begin{aligned} f_n(\eta ) = \frac{1}{n!}\frac{\partial ^{n}f(\eta ; s)}{\partial \eta ^{n}}\bigg |_{s=0},\hspace{0.3cm} g_n(\eta ) = \frac{1}{n!}\frac{\partial ^{n}g(\eta ; s)}{\partial \eta ^{n}}\bigg |_{s=0} \\ \theta _n(\eta ) = \frac{1}{n!}\frac{\partial ^{n}\theta (\eta ; s)}{\partial \eta ^{n}}\bigg |_{s=0},\hspace{0.3cm}\phi _n(\eta ) = \frac{1}{n!}\frac{\partial ^{n}\phi (\eta ; s)}{\partial \eta ^{n}}\bigg |_{s=0} \end{aligned} \end{aligned}$$

It can be noted that in the above series convergence strongly depends upon $\hbar _f$, $\hbar _g$, $\hbar _\theta$ and $\hbar _\phi$.

Assuming that these nonzero auxiliary parameters are chosen so that Eqs. (33–36) converges at $s = 1$. thus we get,

47 $$\begin{aligned}{} & {} f(\eta ) = f_0 + \sum _{n=1}^{\infty }f_n(\eta ) \end{aligned}$$

48 $$\begin{aligned}{} & {} g(\eta ) = g_0 + \sum _{n=1}^{\infty }g_n(\eta ) \end{aligned}$$

49 $$\begin{aligned}{} & {} \theta (\eta ) = \theta _{0} + \sum _{n=1}^{\infty }\theta _n(\eta ) \end{aligned}$$

50 $$\begin{aligned}{} & {} \phi (\eta ) = \phi _0 + \sum _{n=1}^{\infty }\phi _n(\eta ) \end{aligned}$$

Differentiating n-times the deformation Eqs. (33–36) with respect to *s* and then using $s = 0$, we have

51 $$\begin{aligned}{} & {} \pounds _f[f_n(\eta ) - \chi _nf_{n-1}(\eta )] = \hbar _f R_{f,n}(\eta ) \end{aligned}$$

52 $$\begin{aligned}{} & {} \pounds _g[g_n(\eta ) - \chi _ng_{n-1}(\eta )] = \hbar _g R_{g,n}(\eta ) \end{aligned}$$

53 $$\begin{aligned}{} & {} \pounds _\theta [\theta _n(\eta ) - \chi _n\theta _{n-1}(\eta )] = \hbar _\theta R_{\theta ,n}(\eta ) \end{aligned}$$

54 $$\begin{aligned}{} & {} \pounds _\phi [\phi _n(\eta ) - \chi _n\phi _{n-1}(\eta )] = \hbar _\phi R_{\phi ,n}(\eta ) \end{aligned}$$

boundary conditions given are,

55 $$ \begin{aligned}{}&f_n(0) = S, \hspace{0.5cm} f'_n(0) = 1,\hspace{0.5cm} g_n(0) = 0,\hspace{0.5cm} \theta _n(0) = 0, \hspace{0.5cm} \phi _n(0) = 0 \\&f_n(1) = 0.5S_q, \hspace{0.5cm} f'_n(1) = 0,\hspace{0.5cm} g_n(1) =0,\hspace{0.5cm} \theta _n(1) = 1, \hspace{0.5cm} \phi _n(1) = 1  \end{aligned}$$

where

56 $$\begin{aligned}{} & {} \begin{array}{c} R_{f,n}(\eta ) = f^{''''}_{n-1}(\eta )+\sum _{j=0}^{n-1}\bigg (f_{j}(\eta )f^{'''}_{n-j-1}(\eta )-f_{j} (\eta )f^{''}_{n-j-1}(\eta )\bigg )-\dfrac{S_q}{2}\left( 3f^{''}_{n-1}(\eta )+\eta f^{'''}_{n-1}(\eta )\right) -\\ 2\Omega g^{'}_{n-1}(\eta )-M^{2}f^{''}_{n-1}(\eta ), \end{array} \end{aligned}$$

57 $$\begin{aligned}{} & {} \begin{array}{c} R_{g,n}(\eta )= g^{''}_{n-1}(\eta )+\sum _{j=0}^{n-1}\bigg (f_{j}(\eta )g^{'}_{n-j-1}(\eta )-g_{j} (\eta )f^{'}_{n-j-1}(\eta )\bigg )-\dfrac{S_q}{2}\left( 2g_{n-1}(\eta )+ \eta g^{'}_{n-1}(\eta ) \right) +\\ 2 \Omega f^{'}_{n-1}(\eta )-M^{2}g_{n-1}(\eta ) \end{array} \end{aligned}$$

58 $$ \begin{aligned} R_{\theta ,n}(\eta )&=\left( 3+4R_d\right) \theta ^{''}_{n-1}(\eta )+ 3P_r \left( D_u \phi ^{''}_{n-1}(\eta )+ \sum _{j=0}^{n-1}f_{j}(\eta )\theta ^{'}_{n-j-1}(\eta )-\dfrac{S_q}{2}\left( \eta \theta ^{'}_{n-1}(\eta )+2 \theta _{n-1}(\eta ) \right) \right) +\\ {}&3P_rE_c\left( {f^{''}}^2_{n-1}(\eta )+{g^{'}}^2_{n-1}(\eta ) +\delta ^{2}\left( 4{f^{'}}^2_{n-1}(\eta )+g^{2}_{n-1}(\eta )\right) +M^{2} \left( {f^{'}}^2_{n-1}(\eta )+g^{2}_{n-1}(\eta )\right) \right)  \end{aligned}$$

59 $$ \begin{aligned} R_{\phi ,n}(\eta )&= \phi ^{''}_{n-1}(\eta )+S_c \left( \sum _{j=0}^{n-1}f_{j}(\eta )\phi ^{'}_{n-j-1}(\eta )-\dfrac{S_q}{2}\left( 2\phi _{n-1}(\eta )+\eta \phi ^{'}_{n-1}(\eta )\right) +S_r\theta ^{''}_{n-1}(\eta )\right)  \end{aligned}$$

and $$\chi _n = \bigg \{1,\,\, if\,\, n > 1,\,\,\,\,\, and \,\, 0,\,\,if\,\, n = 1$$

Finally, the general solution of Eqs. (51–54) can be written as

60 $$\begin{aligned}{} & {} \begin{aligned} f_n(\eta ) = \int _{0}^{\eta }\int _{0}^{\eta }\int _{0}^{\eta }\int _{0}^{\eta }\hbar _f R_{f,n}(z)dzdzdzdz + \\ \chi _nf_{n-1} + K_{1^*}\eta ^3 + K_{2^*}\eta ^2 + K_{3^*}\eta + K_{4^*} \end{aligned} \end{aligned}$$

61 $$\begin{aligned}{} & {} g_n(\eta ) = \int _{0}^{\eta }\int _{0}^{\eta }\hbar _g R_{g,n}(z)dzdz + \chi _ng_{n-1} + K_{5^*}\eta + K_{6^*} \end{aligned}$$

62 $$\begin{aligned}{} & {} \theta _n(\eta ) = \int _{0}^{\eta }\int _{0}^{\eta }\hbar _\theta R_{\theta ,n}(z)dzdz + \chi _n\theta _{n-1} + K_{7^*}\eta + K_{8^*} \end{aligned}$$

63 $$\begin{aligned}{} & {} \phi _n(\eta ) = \int _{0}^{\eta }\int _{0}^{\eta }\hbar _\phi R_{\phi ,n}(z)dzdz + \chi _n\phi _{n-1} + K_{9^*}\eta + K_{10^*} \end{aligned}$$

and so for $f(\eta )$, $g(\eta )$, $\theta (\eta )$ and $\phi (\eta )$, the exact solution becomes

64 $$ \begin{aligned}{}&f(\eta ) \approx \sum _{m=0}^{n}f_m(\eta ) \\&g(\eta ) \approx \sum _{m=0}^{n}g_m(\eta ) \\&\theta (\eta ) \approx \sum _{m=0}^{n}\theta _m(\eta ) \\&\phi (\eta ) \approx \sum _{m=0}^{n}\phi _m(\eta ). \end{aligned}$$

## Optimal convergence control parameters

It should be noted that the nonzero auxiliary parameters $\hbar _f$, $\hbar _g$, $\hbar _\theta$ and $\hbar _\phi$ contained in the series solutions $(51-54)$, through which the rate of the homotopy series solutions and convergence region can be determined. Average residual error were used to obtain the optimal values of $\hbar _f$, $\hbar _g$, $\hbar _\theta$ and $\hbar _\phi$:

65 $$\begin{aligned}{} & {} \varepsilon ^{f}_{n} = \frac{1}{L + 1}\sum _{j=0}^{L}\bigg [\aleph _f\bigg (\sum _{i=0}^{n}f(\eta ),\sum _{i=0}^{n}g(\eta )\bigg )_{m=j\delta m}\bigg ]^2d\eta \end{aligned}$$

66 $$\begin{aligned}{} & {} \begin{aligned} \varepsilon ^{g}_{n} = \frac{1}{L + 1}\sum _{j=0}^{L}\bigg [\aleph _g\bigg (\sum _{i=0}^{n}f(\eta ), \sum _{i=0}^{n}g(\eta )\bigg )_{m=j\delta m}\bigg ]^2d\eta \end{aligned} \end{aligned}$$

67 $$\begin{aligned}{} & {} \varepsilon ^{\theta }_{n} = \frac{1}{L + 1}\sum _{j=0}^{L}\bigg [\aleph _\theta \bigg (\sum _{i=0}^{n}f(\eta ), \sum _{i=0}^{n}g(\eta ), \sum _{i=0}^{n}\theta (\eta ), \sum _{i=0}^{n}\phi (\eta ) \bigg )_{m=j\delta m}\bigg ]^2d\eta \end{aligned}$$

68 $$\begin{aligned}{} & {} \begin{aligned} \varepsilon ^{\phi }_{n} = \frac{1}{L + 1}\sum _{j=0}^{L}\bigg [\aleph _\phi \bigg (\sum _{i=0}^{n}f(\eta ), \sum _{i=0}^{n}\theta (\eta ),\sum _{i=0}^{n}\phi (\eta )\bigg )_{m=j\delta m}\bigg ]^2d\eta \end{aligned} \end{aligned}$$

Also,

69 $$\begin{aligned} \varepsilon ^{t}_{n} = \varepsilon ^{f}_{n} + \varepsilon ^{g}_{n} + \varepsilon ^{\theta }_{n} + \varepsilon ^{\phi }_{n} \end{aligned}$$

here the total squared residual error is $\varepsilon ^{t}_{n}$. By applying Mathematica package BVPh 2.0 Zhao et al.^37^, we can minimize total average squared residual error. To acquire the local optimal convergence control parameters, the command Minimize was used.

## Error analysis

**Figure 2:**
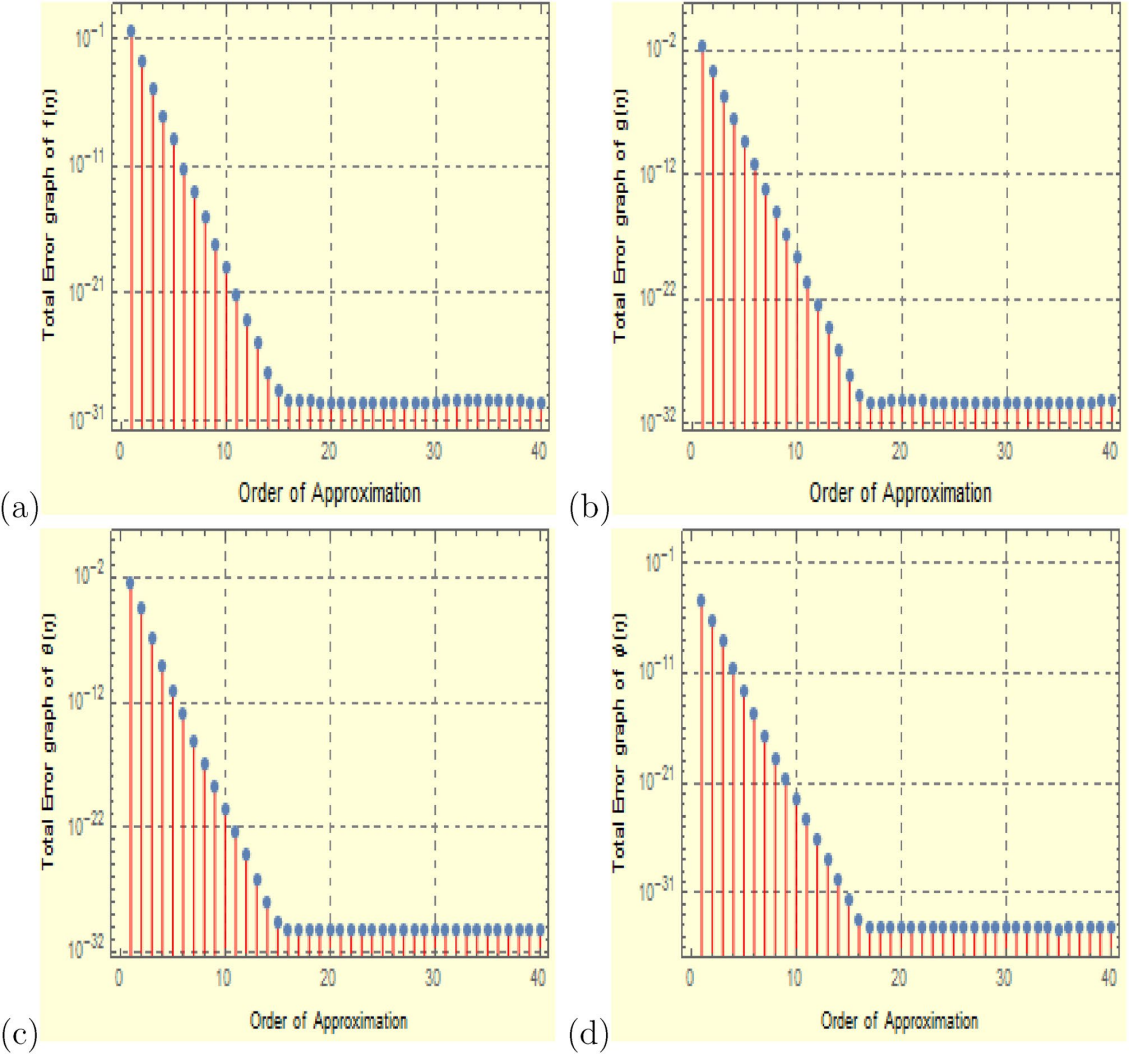
Error profile for $$ f(\eta )$$, $$ g(\eta ) $$, $$ \theta (\eta ) $$, and $$ \phi (\eta ) $$ with $$S_q=1.0$$, $$\Omega =2$$, $$D_u=0.2$$, $$P_r=0.1$$, $$ S_c=0.1 $$, $$ S_r=1 $$, $$ S=0.5$$, $$ M=0.5 $$, $$R_d=1$$, $$E_c=0.2 $$, and $$\delta =0.1$$.

**Figure 3:**
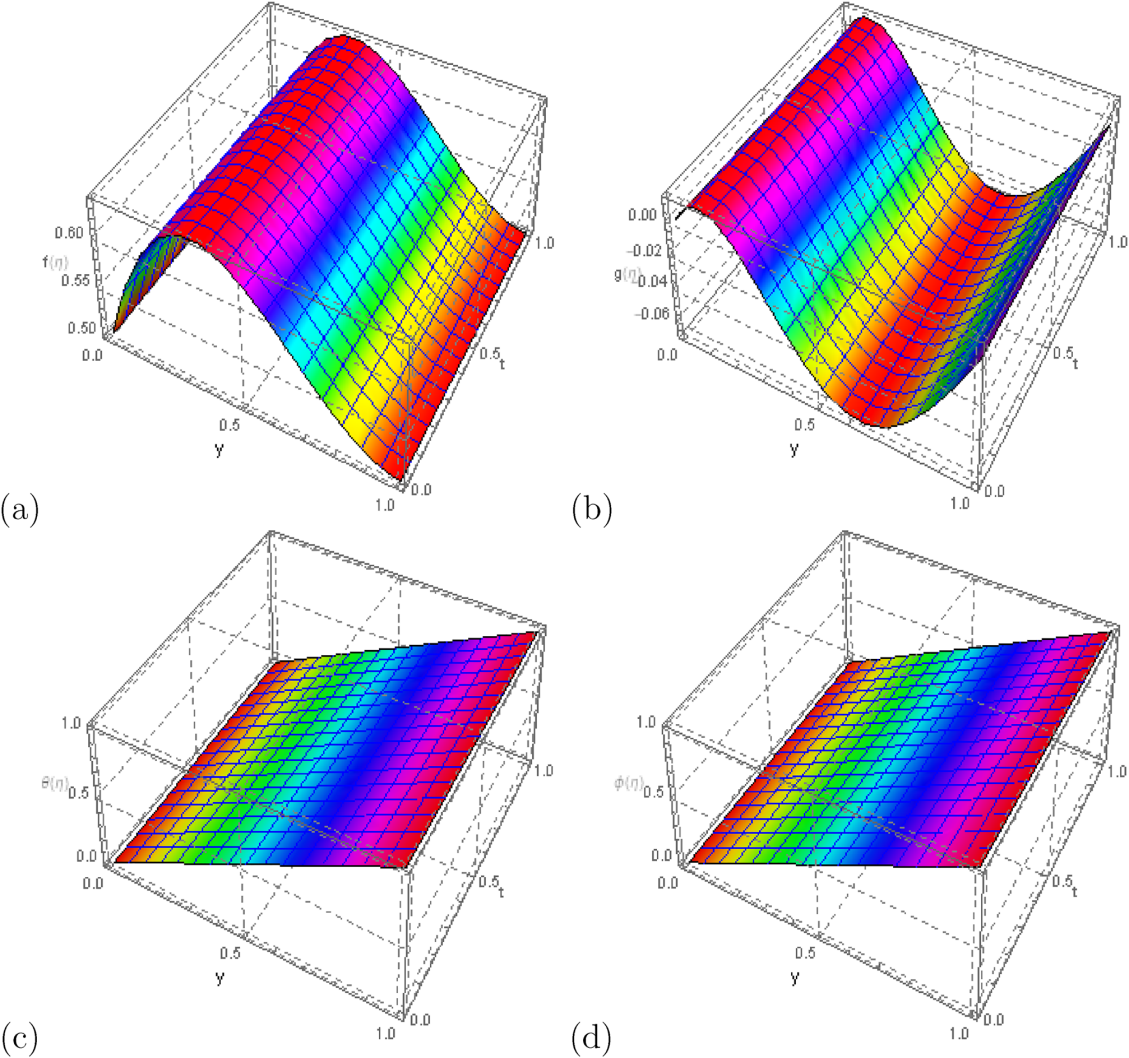
Three dimensional view of the profiles $$ f(\eta )$$, $$ g(\eta ) $$, $$ \theta (\eta )$$, and $$ \phi (\eta ) $$ with $$S_q=1.0$$, $$\Omega =2$$, $$D_u=0.2$$, $$P_r=0.1$$, $$ S_c=0.1 $$, $$ S_r=1 $$, $$ S=0.5$$, $$ M=0.5 $$, $$R_d=1$$, $$E_c=0.2 $$, and $$\delta =0.1$$.

In order to verify that the analysis is acceptable and that the residual error is kept to a minimum, the problem is numerically examined in this section. With a maximum residual error of $$10^{-40}$$, this analysis is also done to determine the viability of the HAM technique using the Mathematica package BVPh 2.0. The analysis is done with $$40\text{th}$$-order approximations. Table 1 and Fig. 2 depict the results of the $$40\text{th}$$-order solutions. At the $$25\text{th}$$-order of approximations, it has been found that the solutions are virtually convergent. Table 2 displays the optimal convergence control parameter values versus various approximation orders with fixed values of $$S_q=1.0$$, $$\Omega =2$$, $$D_u=0.2$$, $$P_r=0.1$$, $$ S_c=0.1 $$, $$ S_r=1 $$, $$ S=0.5$$, $$ M=0.5 $$, $$R_d=1$$, $$E_c=0.2 $$, and $$\delta =0.1$$. The numerical values of velocity, temperature, and concentration distribution are shown in Table 3 and correspond to various values of $$\eta $$. Additionally, it demonstrates the solution’s accuracy by supporting it with the problem’s provided boundary conditions, which can be confirmed by looking at the numerical results at various points. Figure 3 was created to confirm the boundary conditions for $$f(\eta )$$, $$g(\eta )$$, $$\theta (\eta )$$, and $$ \phi (\eta ) $$ in the 3D view. The convergence of homotopy analysis method solution for various order of approximations for the skin friction coefficient, heat flux, and mass flux with $$S_q=1.0$$, $$\Omega =2$$, $$D_u=0.2$$, $$P_r=0.1$$, $$ S_c=0.1 $$, $$ S_r=1 $$, $$ S=0.5$$, $$ M=0.5 $$, $$R_d=1$$, $$E_c=0.2 $$, and $$\delta =0.1$$ is shown in Table 4. These values have been found to be convergent in the $$10\text{th}$$-order of approximations. Tables 5, 6, 7, 8, 9, 10 investigate the numerical results for the the effect of physical parameters on skin friction $$f''(0)$$, $$ -g'(0) $$, $$ -\theta ^{'}(0) $$ and $$-\phi ^{'}(0)$$ with different values of $$S_q,\,\, S,\,\, M,\,\, D_u, \,\, P_r,\,\, E_c$$. Table 5 shows that increasing the squeezing number $$ S_q $$, there is increase in the values of $$f''(0)$$, $$ -g'(0) $$, $$ -\theta ^{'}(0) $$, while the effect on $$-\phi ^{'}(0)$$ is negligible. Further, Table 6 depicts the impact of suction parameter *S* on skin friction showing that the values of $$f''(0)$$, $$ -g'(0) $$, $$ -\theta ^{'}(0) $$ and $$-\phi ^{'}(0)$$ increases with increase in the values of *S*. Increasing values of magnetic number *M* shows a certain increasing effect on $$f''(0)$$ while the effect on $$ -g'(0) $$, $$ -\theta ^{'}(0) $$ and $$ -\phi ^{'}(0)$$ is negligible as can be seen through Table 7. Similarly, from Table 8 it can be observed that Dufour number $$ D_u $$ have a direct impact on both $$ -\theta ^{'}(0) $$ and $$-\phi ^{'}(0)$$. The effect of Prandtl number $$ P_r$$ from Table 9, indicates that $$ -\theta ^{'}(0)$$ increases while $$-\phi ^{'}(0)$$ decreases by increasing the values of $$ P_r$$. Table 10 shows the impact of Eckert number $$ E_c $$ on skin friction showing that the Eckert number $$ E_c $$ have a direct impact on $$ -\theta ^{'}(0)$$ while an inverse impact on $$-\phi ^{'}(0)$$.

**Table 1:** Estimating individual error for different order of approximations with fixed values of $$S_q=1.0$$, $$\Omega =2$$, $$D_u=0.2$$, $$P_r=0.1$$, $$ S_c=0.1 $$, $$ S_r=1 $$, $$ S=0.5$$, $$ M=0.5 $$, $$R_d=1$$, $$E_c=0.2 $$, and $$\delta =0.1$$.

*m*	$\varepsilon _m^f$	$\varepsilon _m^g$	$\varepsilon _m^{\theta }$	$\varepsilon _m^{\phi }$
1	0.34078	0.0214375	0.00306542	0.0000362193
5	1.31385 $\times 10^{-9}$	4.69349 $\times 10^{-10}$	8.19172$\times 10^{-12}$	1.95769$\times 10^{-13}$
10	1.24169$\times 10^{-19}$	2.19766$\times 10^{-19}$	3.13303$\times 10^{-21}$	3.46306 $\times 10^{-23}$
15	1.9463$\times 10^{-29}$	9.53779 $\times 10^{-29}$	2.27437$\times 10^{-30}$	2.41344 $\times 10^{-32}$
20	2.95105$\times 10^{-30}$	6.59002$\times 10^{-31}$	7.44284$\times 10^{-31}$	8.3872$\times 10^{-35}$
25	2.98353$\times 10^{-30}$	6.18862$\times 10^{-31}$	7.44284$\times 10^{-31}$	7.35201$\times 10^{-35}$
30	2.97169$\times 10^{-30}$	6.18015$\times 10^{-31}$	7.44284$\times 10^{-31}$	6.48534$\times 10^{-35}$
35	3.33562$\times 10^{-30}$	6.06028$\times 10^{-31}$	7.44284$\times 10^{-31}$	5.32979$\times 10^{-35}$
40	2.95894$\times 10^{-30}$	6.55782$\times 10^{-31}$	7.44284$\times 10^{-31}$	6.48534$\times 10^{-35}$

**Table 2:** Optimal values of convergence control parameters in comparison different orders of approximation with $$S_q=1.0$$, $$\Omega =2$$, $$D_u=0.2$$, $$P_r=0.1$$, $$ S_c=0.1 $$, $$ S_r=1 $$, $$ S=0.5$$, $$ M=0.5 $$, $$R_d=1$$, $$E_c=0.2 $$, and $$\delta =0.1$$.

Order	$h_f$	$h_g$	$h_{\theta }$	$h_{\phi }$	$\varepsilon _m^t$
2	− 1.0838	− 0.866062	− 0.144849	− 1.08755	0.00120324
3	− 1.08386	− 0.879546	− 0.141488	− 1.07912	8.79403$\times 10^{-6}$
4	− 1.08172	− 0.888358	− 0.144455	− 0.983393	1.3028$\times 10^{-7}$
5	− 0.928336	− 0.996524	− 0.139297	− 1.06943	6.89601$\times 10^{-8}$
6	− 1.0729	− 0.892665	− 0.138309	− 1.03479	$-$4.36697$\times 10^{-12}$
7	− 1.01666	− 0.884029	− 0.130717	− 1.08066	1.25908$\times 10^{-11}$

**Table 3:** Estimated values for velocity, temperatur and concentration profiles versus different values of $$ \eta $$ with $$S_q=1.0$$, $$\Omega =2$$, $$D_u=0.2$$, $$P_r=0.1$$, $$ S_c=0.1 $$, $$ S_r=1 $$, $$ S=0.5$$, $$ M=0.5 $$, $$R_d=1$$, $$E_c=0.2 $$, and $$\delta =0.1$$.

$\eta$	$f(\eta )$	$g(\eta )$	$\theta (\eta )$	$\phi (\eta )$
0.	0.5	0.	0.	0.
0.1	0.579075	0.0128061	0.102072	0.10002
0.2	0.622373	0.00166006	0.202843	0.199745
0.3	0.638065	$-$0.0201089	0.302812	0.299232
0.4	0.633284	$-$0.0429745	0.402334	0.39858
0.5	0.614376	$-$0.0604795	0.501664	0.497916
0.6	0.587107	$-$0.0686805	0.600986	0.597389
0.7	0.556861	$-$0.0657599	0.700426	0.697162
0.8	0.528817	$-$0.0517904	0.800064	0.797406
0.9	0.508111	$-$0.0286392	0.899929	0.898294
1.	0.5	3.1428$\times 10^{-16}$	1.	1.

**Table 4:** The convergence of HAM solution for different orders of approximation for $$ f''(0) $$, $$ -g'(0) $$, $$ -\theta ^{'}(0) $$ and $$ -\phi '(0) $$ when $$S_q=1.0$$, $$\Omega =2$$, $$D_u=0.2$$, $$P_r=0.1$$, $$ S_c=0.1 $$, $$ S_r=1 $$, $$ S=0.5$$, $$ M=0.5 $$, $$R_d=1$$, $$E_c=0.2 $$, and $$\delta =0.1$$.

*m*	$f^{''}(0)$	$-g^{'}(0)$	$- \theta ^{'}(0)$	$-\phi ^{'}(0)$
1	7.4286	$-$2.9362	$-$0.8778	$-$0.9553
5	7.7033	$-$3.0253	$-$0.8694	$-$0.9697
10	7.7034	$-$3.0251	$-$0.8695	$-$0.9697
15	7.7032	$-$3.0251	$-$0.8695	$-$0.9697
20	7.7032	$-$3.0251	$-$0.8695	$-$0.9697
25	7.7032	$-$3.0251	$-$0.8695	$-$0.9697
30	7.7032	$-$3.0251	$-$0.8695	$-$0.9697
35	7.7032	$-$3.0251	$-$0.8695	$-$0.9697
40	7.7032	$-$3.0251	$-$0.8695	$-$0.9697

**Table 5:** Effect of $$S_q$$ on $$f^{''}(0)$$, $$-g^{'}(0)$$, $$-\theta ^{'}(0)$$ and $$-\phi ^{'}(0)$$ with fixed values of $$\Omega =2$$, $$D_u=0.2$$, $$P_r=0.1$$, $$ S_c=0.1 $$, $$ S_r=1 $$, $$ S=0.5$$, $$ M=0.5 $$, $$R_d=1$$, $$E_c=0.2 $$, and $$\delta =0.1$$.

$S_q$	$f^{''}(0)$	$-g^{'}(0)$	$-\theta ^{'}(0)$	$-\phi ^{'}(0)$
− 0.1	$-$7.89	1.06	$-$1.10	$-$1.01
− 0.5	$-$9.08	1.71	$-$1.13	$-$1.02
− 1.0	$-$10.53	2.71	$-$1.19	$-$1.02
− 1.5	$-$11.96	3.98	$-$1.26	$-$1.02

**Table 6:** Effect of *S* on $$f^{''}(0)$$, $$-g^{'}(0)$$, $$-\theta ^{'}(0)$$ and $$-\phi ^{'}(0)$$ with fixed values of $$\Omega =2$$, $$D_u=0.2$$, $$P_r=0.1$$, $$ S_c=0.1 $$, $$ S_r=1 $$, $$ S_q=1.0$$, $$ M=0.5 $$, $$R_d=1$$, $$E_c=0.2 $$, and $$\delta =0.1$$.

*S*	$f^{''}(0)$	$-g^{'}(0)$	$-\theta ^{'}(0)$	$-\phi ^{'}(0)$
0.1	$-$1.80	$-$1.03	$-$1.0	$-$0.99
0.5	$-$4.51	$-$0.30	$-$1.03	$-$1.0
1.0	$-$8.19	0.92	$-$1.09	$-$1.01
1.5	$-$12.21	2.58	$-$1.19	$-$1.02

**Table 7:** Effect of *M* on $$f^{''}(0)$$, $$-g^{'}(0)$$, $$-\theta ^{'}(0)$$ and $$-\phi ^{'}(0)$$ with fixed values of $$\Omega =2$$, $$D_u=0.2$$, $$P_r=0.1$$, $$ S_c=0.1 $$, $$ S_r=1 $$, $$ S_q=1.0$$, $$ S=0.5 $$, $$R_d=1$$, $$E_c=0.2 $$, and $$\delta =0.1$$.

*M*	$f^{''}(0)$	$-g^{'}(0)$	$-\theta ^{'}(0)$	$-\phi ^{'}(0)$
0.1	$-$4.48	$-$0.30	$-$1.03	$-$1.0
0.5	$-$4.51	$-$0.30	$-$1.03	$-$1.0
1	$-$4.61	$-$0.31	$-$1.03	$-$1.0
1.5	$-$4.76	$-$0.31	$-$1.03	$-$1.0

**Table 8:** Effect of $$D_u$$ on $$f^{''}(0)$$, $$-g^{'}(0)$$, $$-\theta ^{'}(0)$$ and $$-\phi ^{'}(0)$$ with fixed values of $$\Omega =2$$, $$E_c=0.2$$, $$P_r=0.1$$, $$ S_c=0.1 $$, $$ S_r=1 $$, $$ S_q=1.0$$, $$ S=0.5 $$, $$R_d=1$$, $$M=0.5 $$, and $$\delta =0.1$$.

$D_u$	$f^{''}(0)$	$-g^{'}(0)$	$-\theta ^{'}(0)$	$-\phi ^{'}(0)$
0.1	$-$4.51	$-$0.30	$-$1.03	$-$1.0
1.0	$-$4.51	$-$0.30	$-$1.03	$-$1.0
2.0	$-$4.51	$-$0.30	$-$1.03	$-$1.0
3.0	$-$4.51	$-$0.30	$-$1.03	$-$1.0

**Table 9:** Effect of $$P_r$$ on $$f^{''}(0)$$, $$-g^{'}(0)$$, $$-\theta ^{'}(0)$$ and $$-\phi ^{'}(0)$$ with fixed values of $$\Omega =2$$, $$E_c=0.2$$, $$D_u=0.2$$, $$ S_c=0.1 $$, $$ S_r=1 $$, $$ S_q=1.0$$, $$ S=0.5 $$, $$R_d=1$$, $$M=0.5 $$, and $$\delta =0.1$$.

$P_r$	$f^{''}(0)$	$-g^{'}(0)$	$-\theta ^{'}(0)$	$-\phi ^{'}(0)$
1.0	$-$4.51	$-$0.30	$-$1.296	$-$0.98
2.0	$-$4.51	$-$0.30	$-$1.597	$-$0.94
3.0	$-$4.51	$-$0.30	$-$1.90	$-$0.91
4.0	$-$4.51	$-$0.30	$-$2.21	$-$0.88

**Table 10:** Effect of $$E_c$$ on $$f^{''}(0)$$, $$-g^{'}(0)$$, $$-\theta ^{'}(0)$$ and $$-\phi ^{'}(0)$$ with fixed values of $$\Omega =2$$, $$P_r=0.1$$, $$D_u=0.2$$, $$ S_c=0.1 $$, $$ S_r=1 $$, $$ S_q=1.0$$, $$ S=0.5 $$, $$R_d=1$$, $$M=0.5 $$, and $$\delta =0.1$$.

$E_c$	$f^{''}(0)$	$-g^{'}(0)$	$-\theta ^{'}(0)$	$-\phi ^{'}(0)$
0.5	$-$4.51	$-$0.30	$-$1.07	$-$0.998
1.0	$-$4.51	$-$0.30	$-$1.14	$-$0.991
1.5	$-$4.51	$-$0.30	$-$1.21	$-$0.984
2.0	$-$4.51	$-$0.30	$-$1.28	$-$0.98

## Results and discussion

The main objective of this study is to investigate the influence of Joule heating and viscous dissipation on the flow of a viscous fluid between squeezing plates under the presence of a uniform magnetic field. The analysis also considers heat and mass transfer through Soret and Dufour effects. To accomplish this, the governing system of equations is transformed into a nonlinear coupled system of ordinary differential equations using appropriate similarity transformations. The analytical solution is then obtained using the homotopy analysis method. The study further explores the impact of various physical parameters, such as the squeezing variable $S_q$, suction variable *S*, magnetic variable *M*, rotation parameter $\Omega$, Eckert number $E_c$, Soret number $S_r$, Dufour number $D_u$, thermal radiation parameter $R_d$, Schmidt number $S_c$, and Prandtl number $P_r$, on the velocity components $f(\eta )$, $f'(\eta )$, and $g(\eta )$, temperature $\theta (\eta )$, and concentration distribution $\phi (\eta )$.

**Figure 4:**
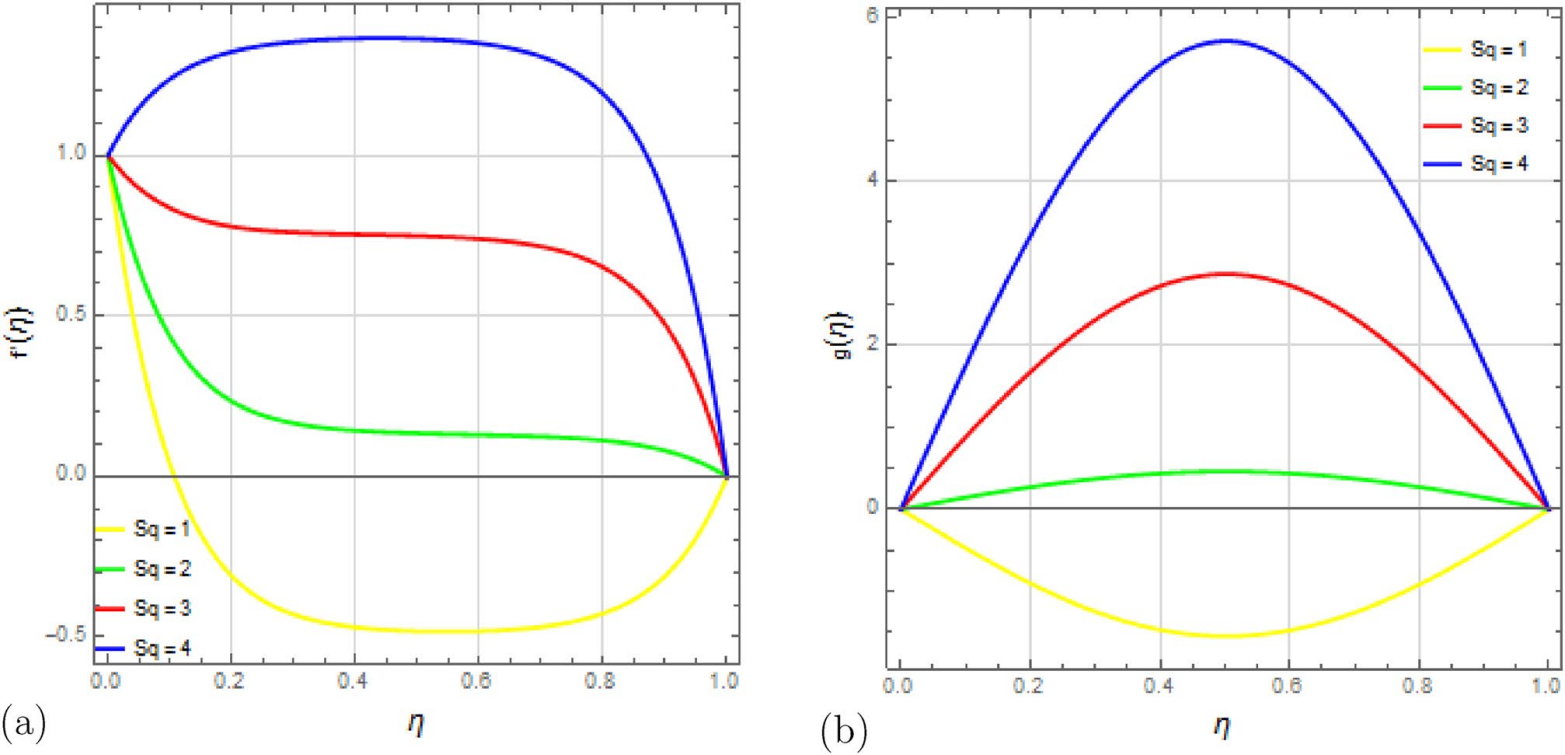
Impact of squeezing number $$ S_q $$ on velocity profiles $$f'(\eta )$$ and $$g(\eta )$$ with $$\Omega =1$$, $$D_u=0.5$$, $$P_r=0.5$$, $$ S_c=0.9 $$, $$ S_r=0.2 $$, $$ S=0.8$$, $$ M=10 $$, $$R_d=1$$, $$E_c=0.2 $$ and $$\delta =0.1$$.

**Figure 5:**
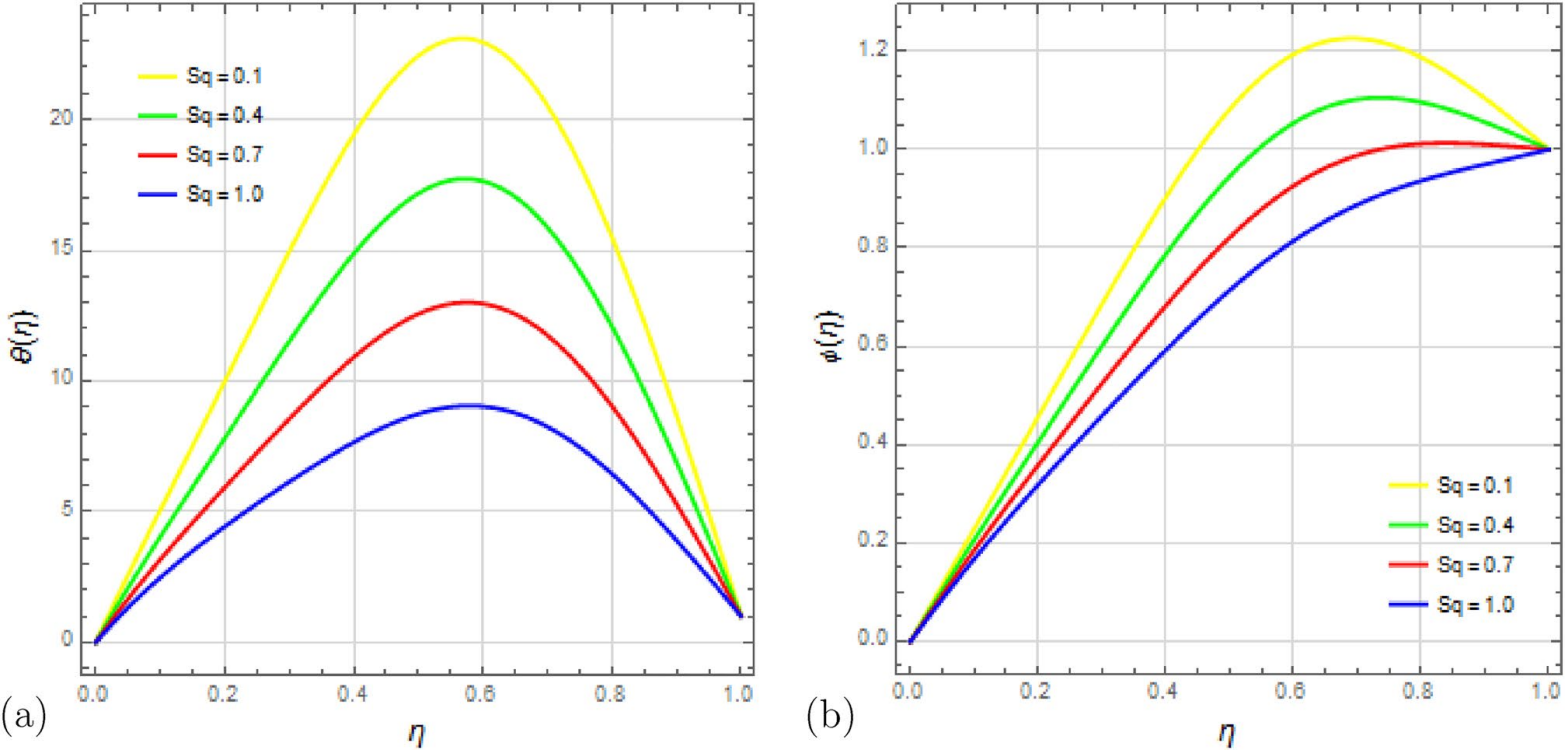
Impact of squeezing number $$ S_q $$ on $$ \theta (\eta ) $$ and $$\phi (\eta )$$ with $$\Omega =1$$, $$D_u=0.5$$, $$P_r=0.5$$, $$ S_c=0.9 $$, $$ S_r=0.2 $$, $$ S=0.8$$, $$ M=10 $$, $$R_d=1$$, $$E_c=0.2 $$ and $$\delta =0.1$$.

The effect of the squeezing parameter $S_q$ on the flow variables is demonstrated in Figs. 4 and 5. Figure 4a shows that the squeezing number $S_q$ influences the velocity distribution $f'(\eta )$, with a degradation near the permeable plate due to stronger suction effects. The pressure generated by the motion of the top plate towards the stretched permeable plate enhances the flow, easing the mass conservation restriction near the top plate. Figure 4b illustrates that increasing $S_q$ results in higher velocity distribution $g(\eta )$, particularly in the middle region of the channel. The impact of squeezing variable $S_q$ on temperature distribution $\theta (\eta )$ and concentration distribution $\phi (\eta )$ is presented in Fig. 5a,b respectively, showing that higher squeezing number $S_q$ leads to lower temperature and concentration distributions.

**Figure 6:**
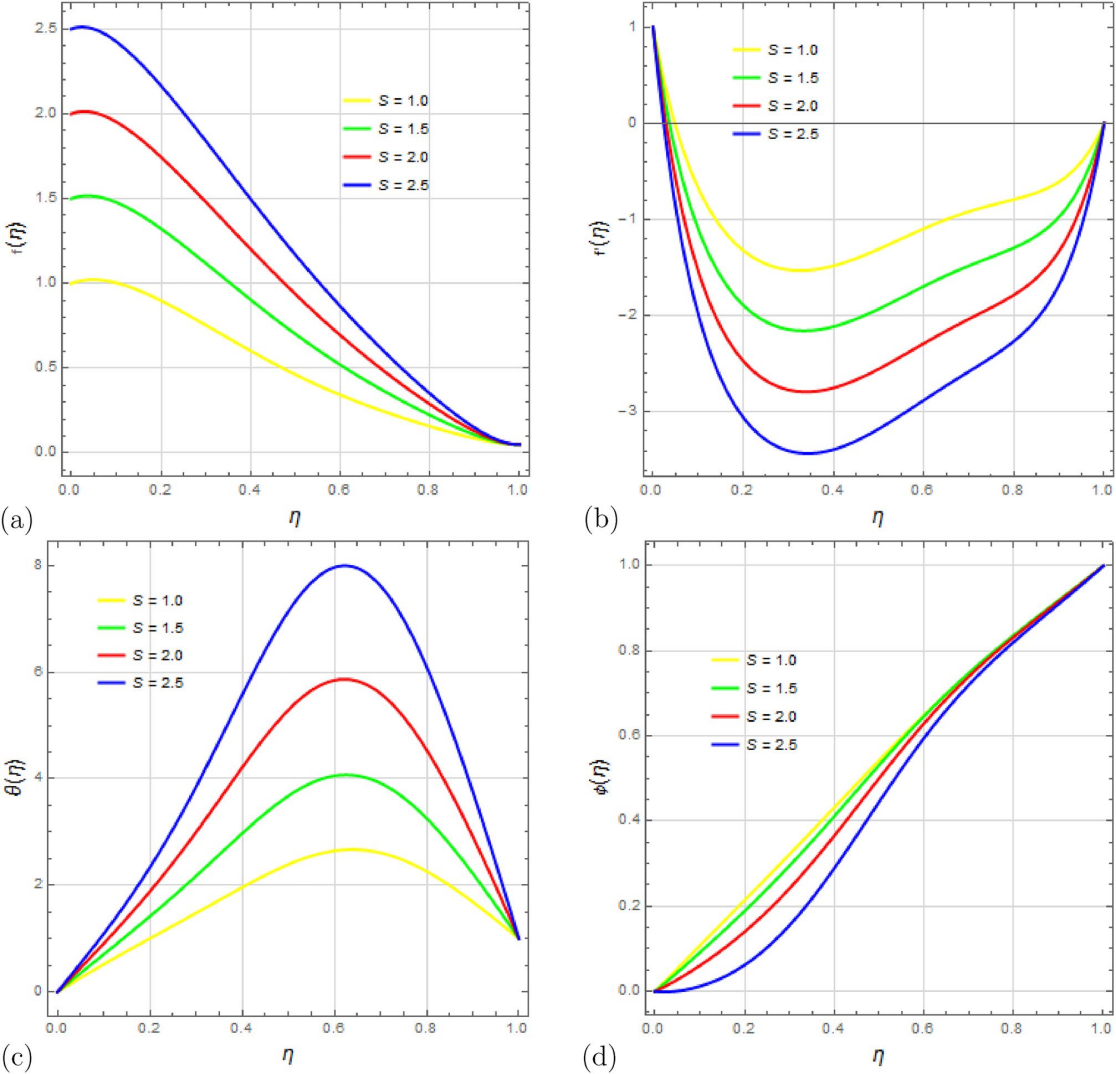
Impact of suction parameter *S* on profiles $$ f(\eta ) $$, $$ f'(\eta ) $$, $$\theta (\eta )$$ and $$ \phi (\eta ) $$ with $$\Omega =0.25$$, $$D_u=0.1$$, $$P_r=0.25$$, $$ S_c=0.5 $$, $$ S_r=0.25 $$, $$ S_q=0.1$$, $$ M=10 $$, $$R_d=0.1$$, $$E_c=0.1 $$ and $$\delta =0.1$$.

Next, the effect of the suction parameter *S* on the flow variables is studied in Fig. 6a–d. Figure 6a indicates that as suction increases, the velocity component $f(\eta )$ grows, and the velocity profile narrows around the bottom plate. Figure 6b shows that stronger suction leads to reduced velocity distribution $f'(\eta )$ and the development of reverse flow, especially near the top plate. Furthermore, Fig. 6c demonstrates that increasing the suction parameter *S* results in a higher temperature distribution $\theta (\eta )$. The impact of suction parameter *S* on concentration $\phi (\eta )$ is shown in Fig. 6d, revealing that higher suction results in lower concentration.

**Figure 7:**
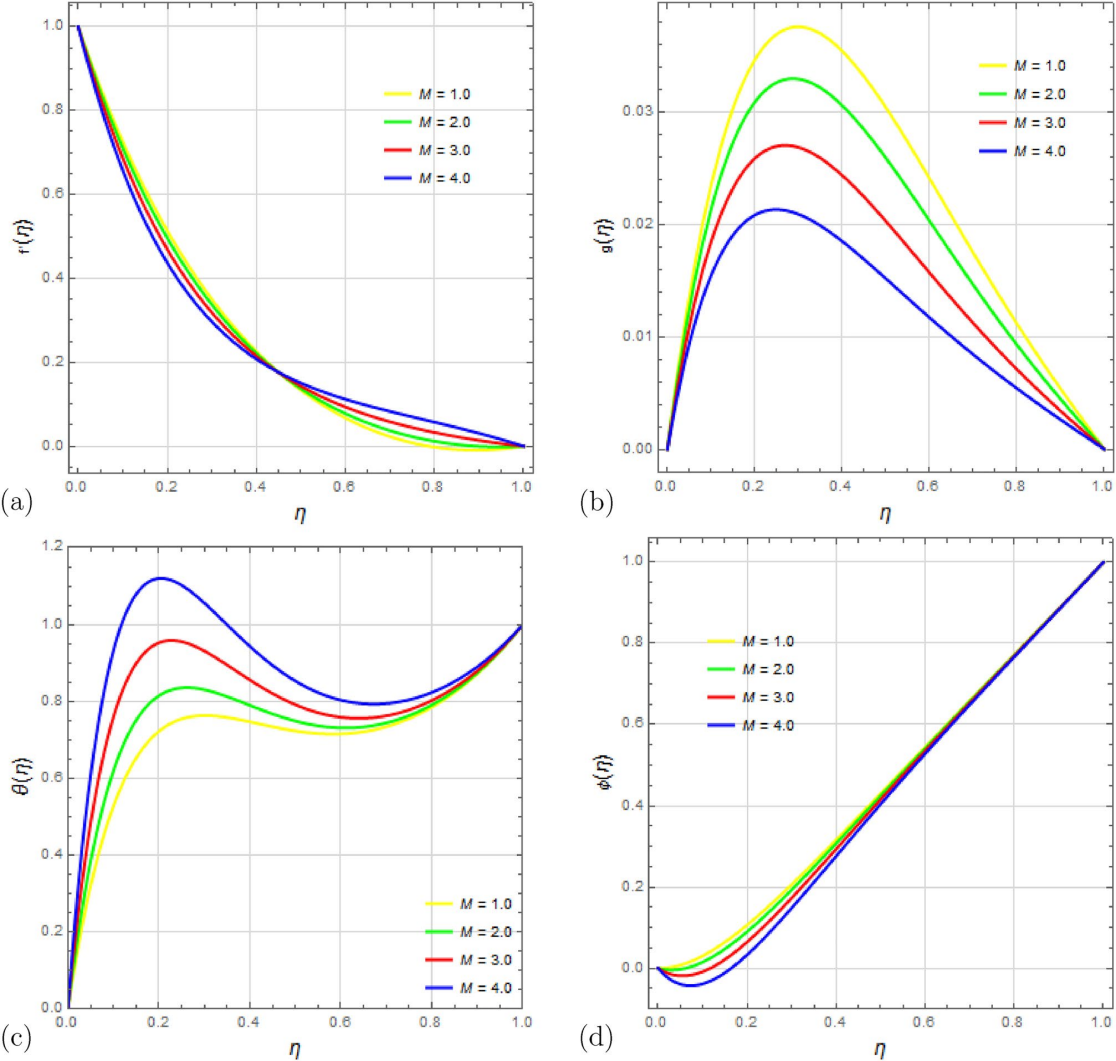
Impact of magnetic number *M* on profiles $$f'(\eta )$$, $$g(\eta )$$, $$\theta (\eta )$$ and $$\phi (\eta )$$ with $$\Omega =1$$, $$D_u=1$$, $$P_r=2.5$$, $$ S_c=0.5 $$, $$ S_r=0.5 $$, $$ S=1$$, $$ S_q=2.5 $$, $$R_d=0.1$$, $$E_c=1.5 $$ and $$\delta =0.1$$.

The influence of the magnetic number *M* on the flow variables is explored in Fig. 7a–d. Figure 7a shows that the velocity component $f'(\eta )$ initially declines with increasing magnetic number *M*, but starts to grow as $\eta \rightarrow 1$. Physically, raising magnetic number *M* decreases the velocity and its gradient. Figure 7b reveals that an increment in *M* leads to a lower value of velocity component $g(\eta )$. Figure 7c,d display the effect of *M* on temperature distribution $\theta (\eta )$ and concentration distribution $\phi (\eta )$, respectively. Temperature increases with the magnetic number *M*, while concentration decreases. This is due to the resistive force (Lorentz force) generated by a higher magnetic field *M*, which opposes the flow and slows down the fluid particles, resulting in decreased velocity and increased temperature, but decreased concentration.

**Figure 8:**
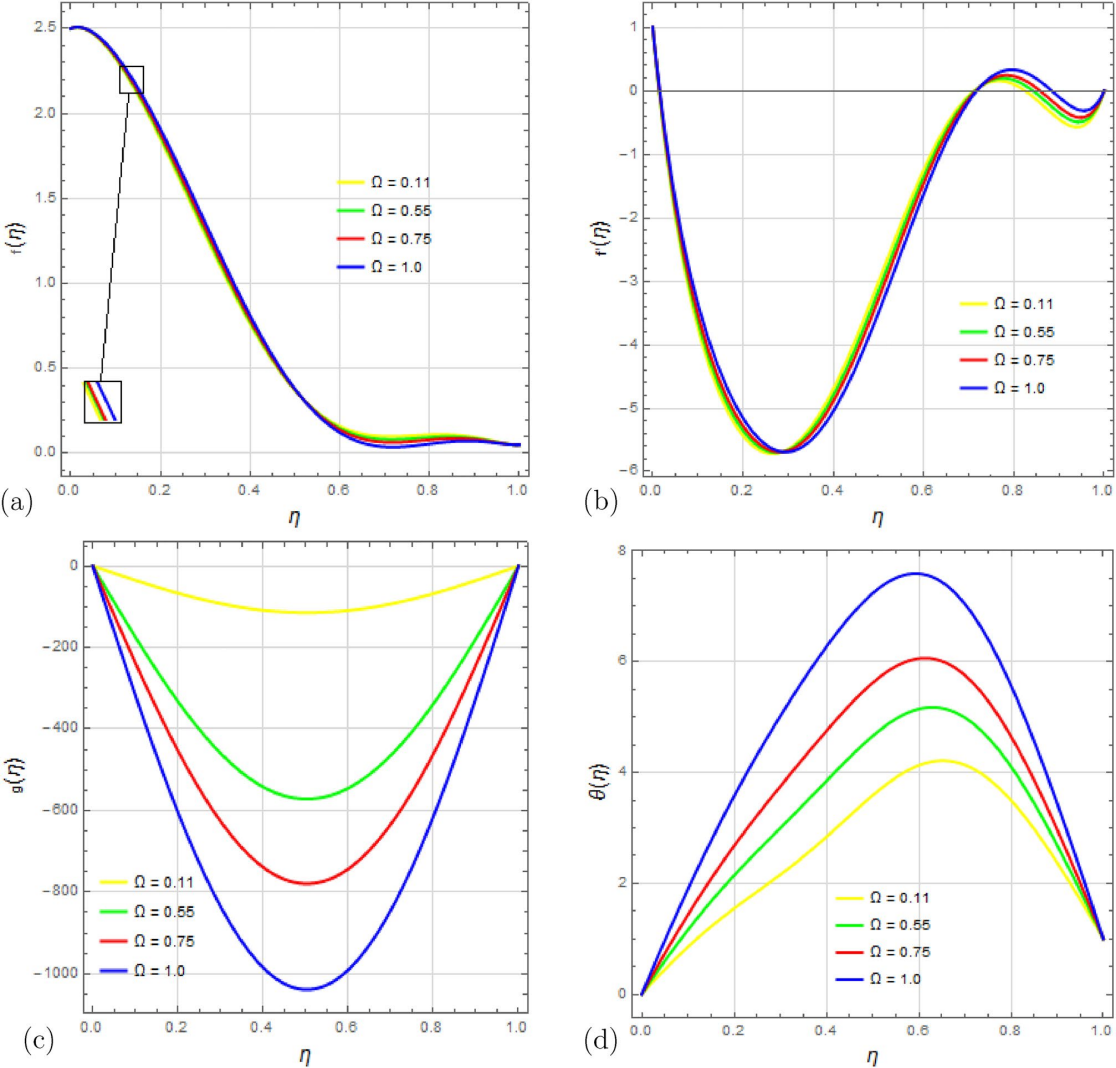
Impact of rotation parameter $$ \Omega $$ on profiles $$f(\eta )$$, $$f'(\eta )$$, $$g(\eta )$$ and $$\theta (\eta )$$ with $$S_q=0.1$$, $$D_u=0.1$$, $$P_r=0.1$$, $$ S_c=0.1 $$, $$ S_r=0.02 $$, $$ S=2.5$$, $$ M=15 $$, $$R_d=0.1$$, $$E_c=0.1 $$ and $$\delta =0.1$$.

**Figure 9:**
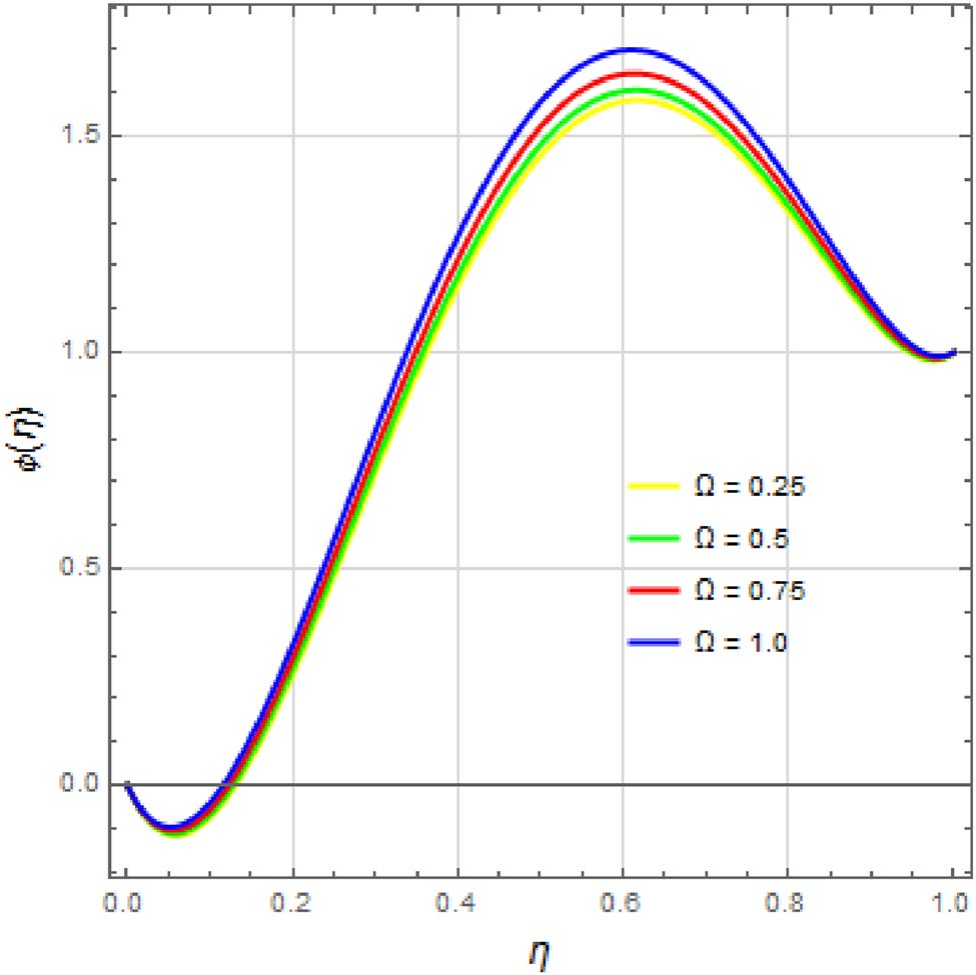
Impact of rotation parameter $$ \Omega $$ on concentration profile $$\phi (\eta )$$ with $$S_q=20$$, $$D_u=0.1$$, $$P_r=0.01$$, $$ S_c=2.5 $$, $$ S_r=0.025 $$, $$ S=5$$, $$ M=10 $$, $$R_d=0.1$$, $$E_c=20 $$ and $$\delta =0.1$$.

**Figure 10:**
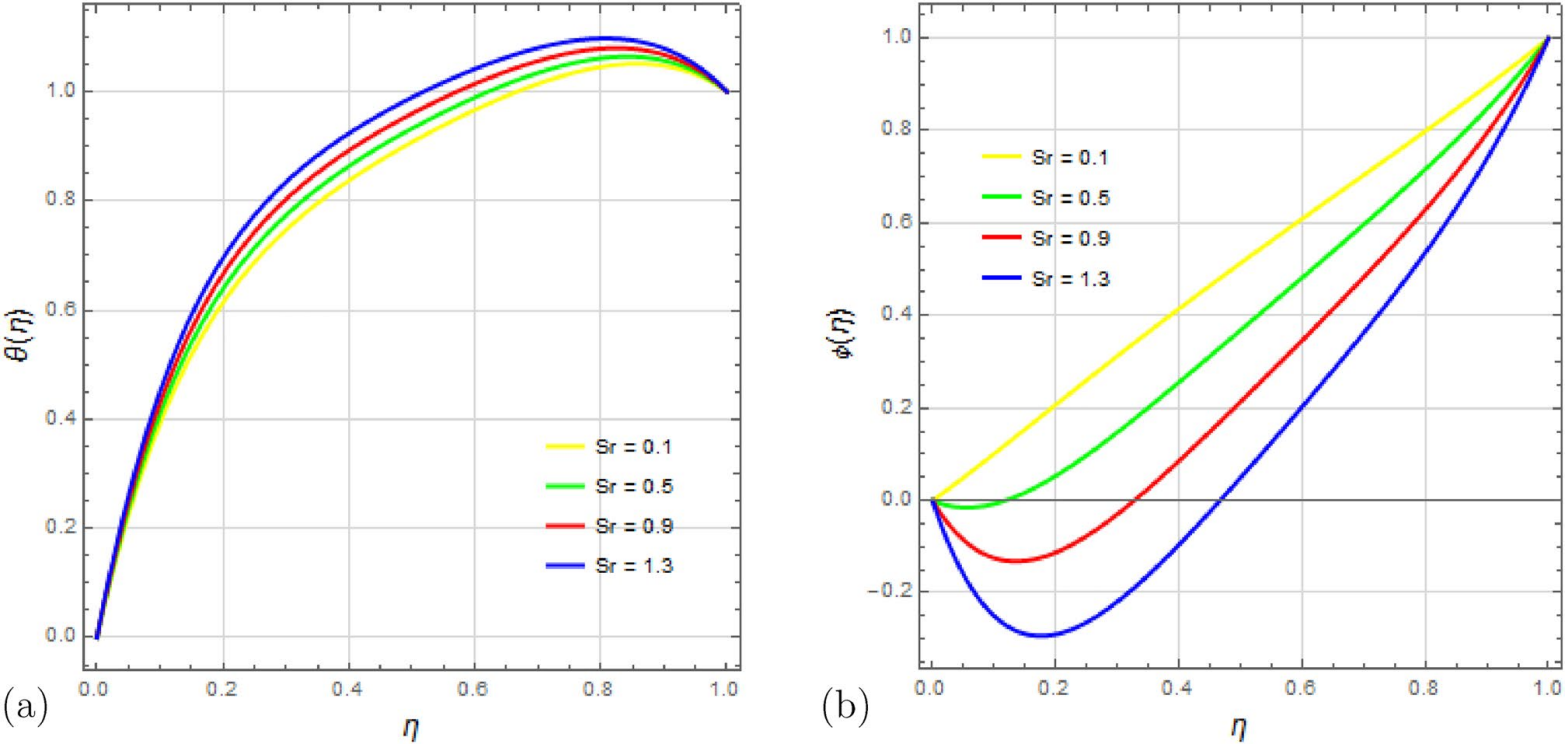
Impact of Soret number $$ S_r $$ on temperature profile $$\theta (\eta )$$ and concentration profile $$\phi (\eta )$$ with $$\Omega =0.1$$, $$D_u=0.1$$, $$P_r=2$$, $$ S_c=0.9 $$, $$ S_q=0.25 $$, $$ S=1$$, $$ M=1.5 $$, $$R_d=0.1$$, $$E_c=0.1 $$ and $$\delta =0.1$$.

**Figure 11:**
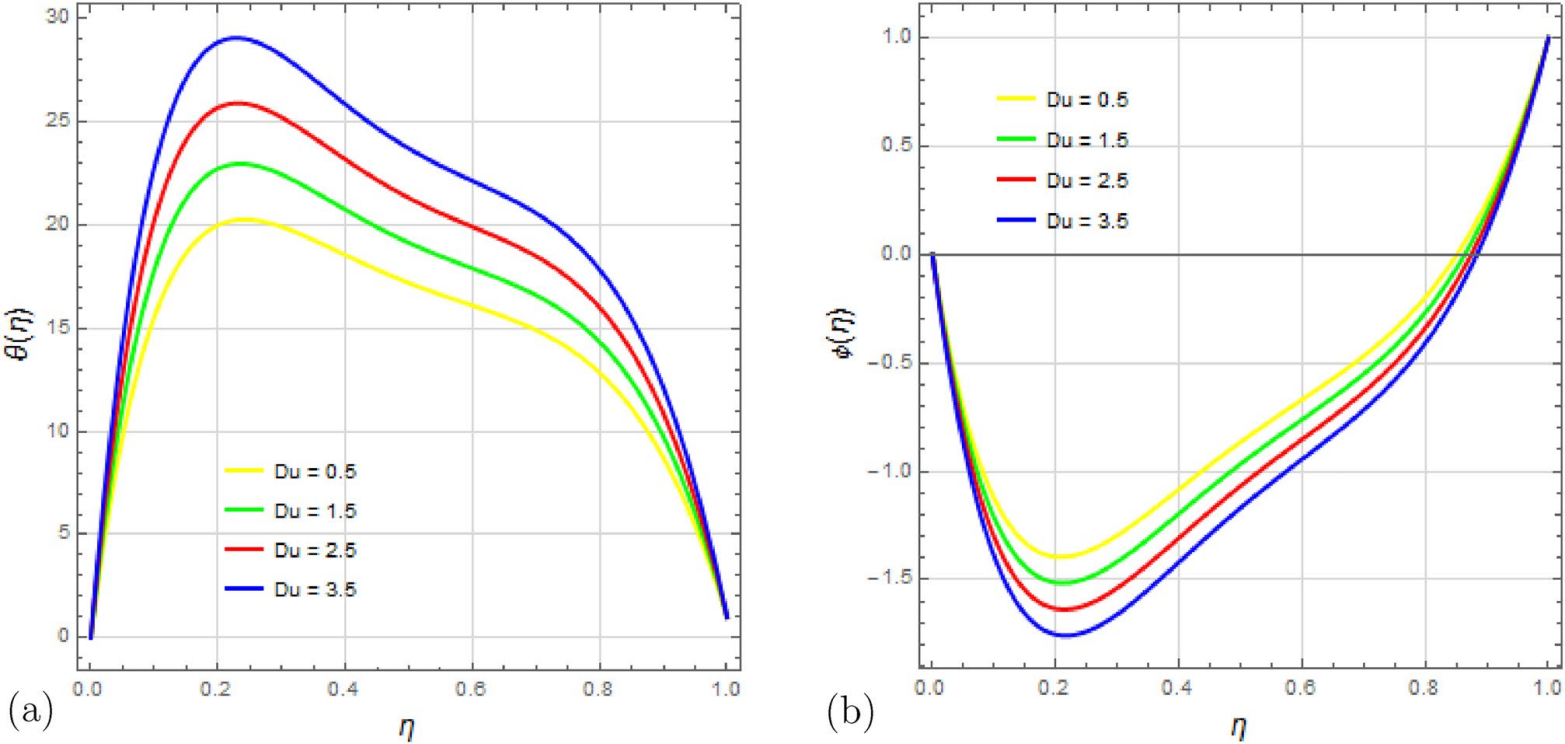
Impact of Dufour number $$ D_u $$ on temperature profile $$\theta (\eta )$$ and concentration profile $$\phi (\eta )$$ with $$\Omega =0.1$$, $$S_q=0.5$$, $$P_r=2$$, $$ S_c=0.25 $$, $$ S_r=0.4 $$, $$ S=2$$, $$ M=0.5 $$, $$R_d=0.1$$, $$E_c=2.5 $$ and $$\delta =0.1$$.

**Figure 12:**
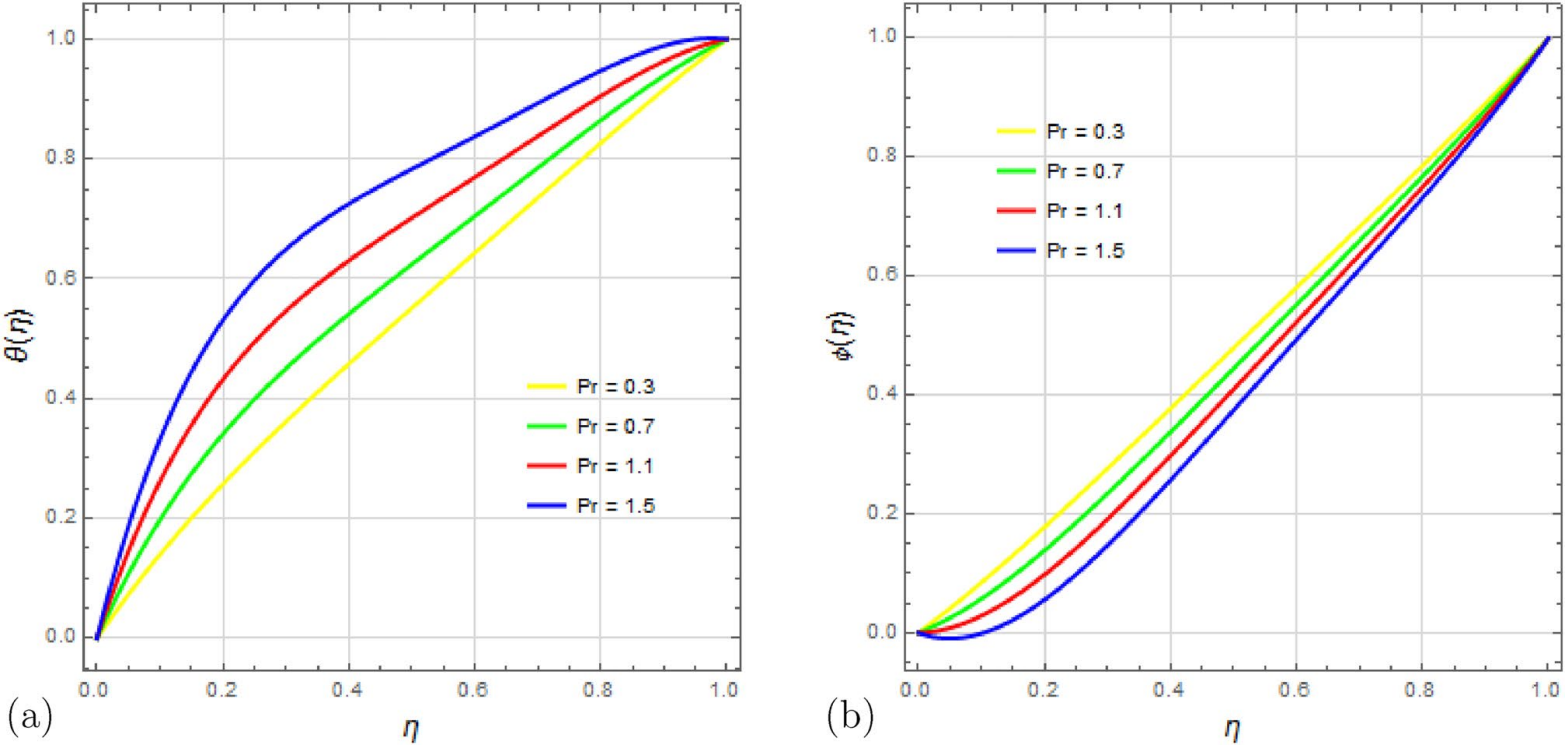
Impact of Prandtl number $$ P_r $$ on temperature profile $$\theta (\eta )$$ and concentration profile $$\phi (\eta )$$ with $$\Omega =1$$, $$D_u=0.5$$, $$S_q=0.5$$, $$ S_c=0.5 $$, $$ S_r=1.5 $$, $$ S=0.5$$, $$ M=0.5 $$, $$R_d=0.5$$, $$E_c=0.5 $$ and $$\delta =0.1$$.

**Figure 13:**
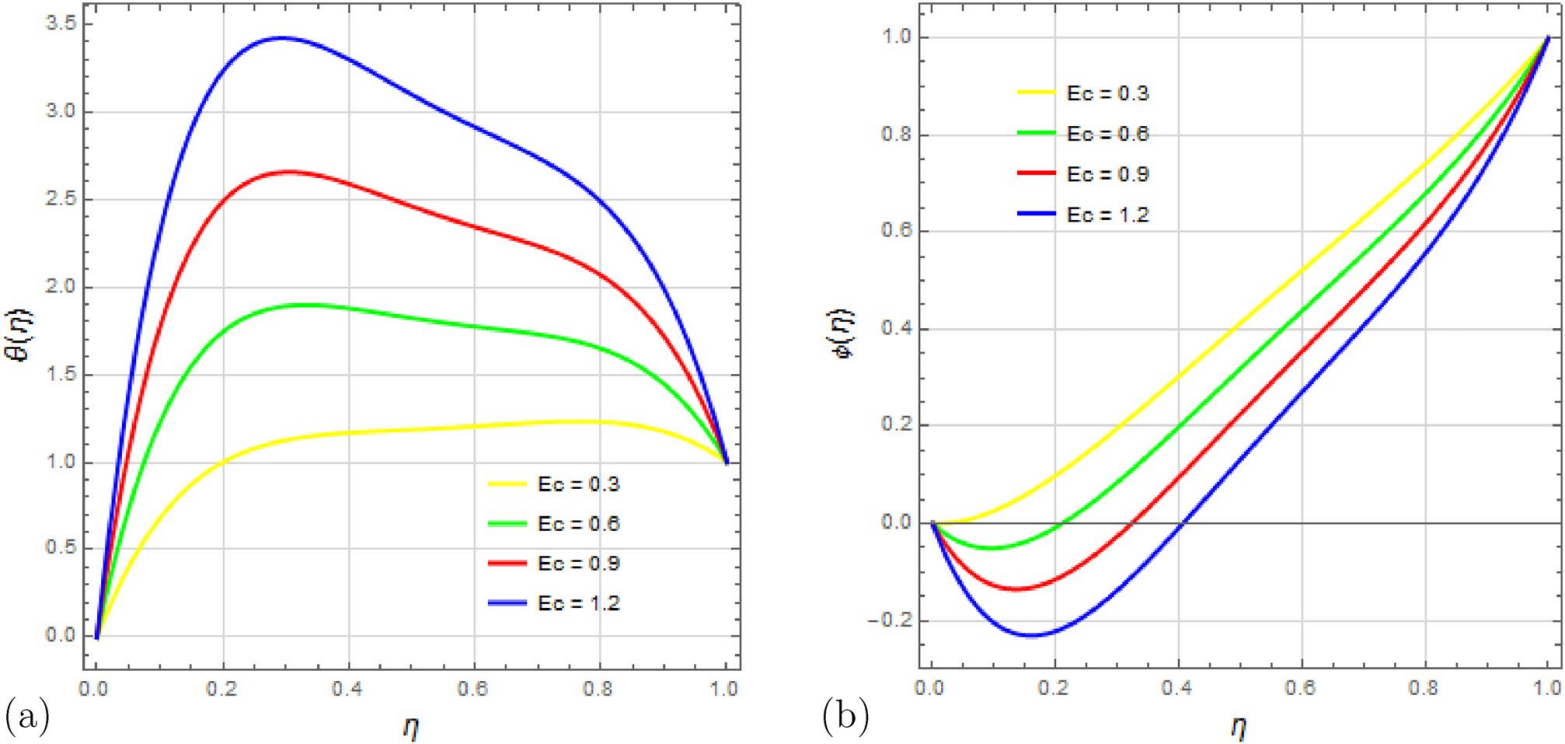
Impact of Eckert number $$ E_c $$ on temperature profile $$\theta (\eta )$$ and concentration profile $$\phi (\eta )$$ with $$\Omega =1$$, $$D_u=1$$, $$S_q=0.5$$, $$ S_c=0.5 $$, $$ S_r=0.5 $$, $$ S=1$$, $$ M=0.5 $$, $$R_d=0.5$$, $$P_r=2.5 $$ and $$\delta =0.1$$.

**Figure 14:**
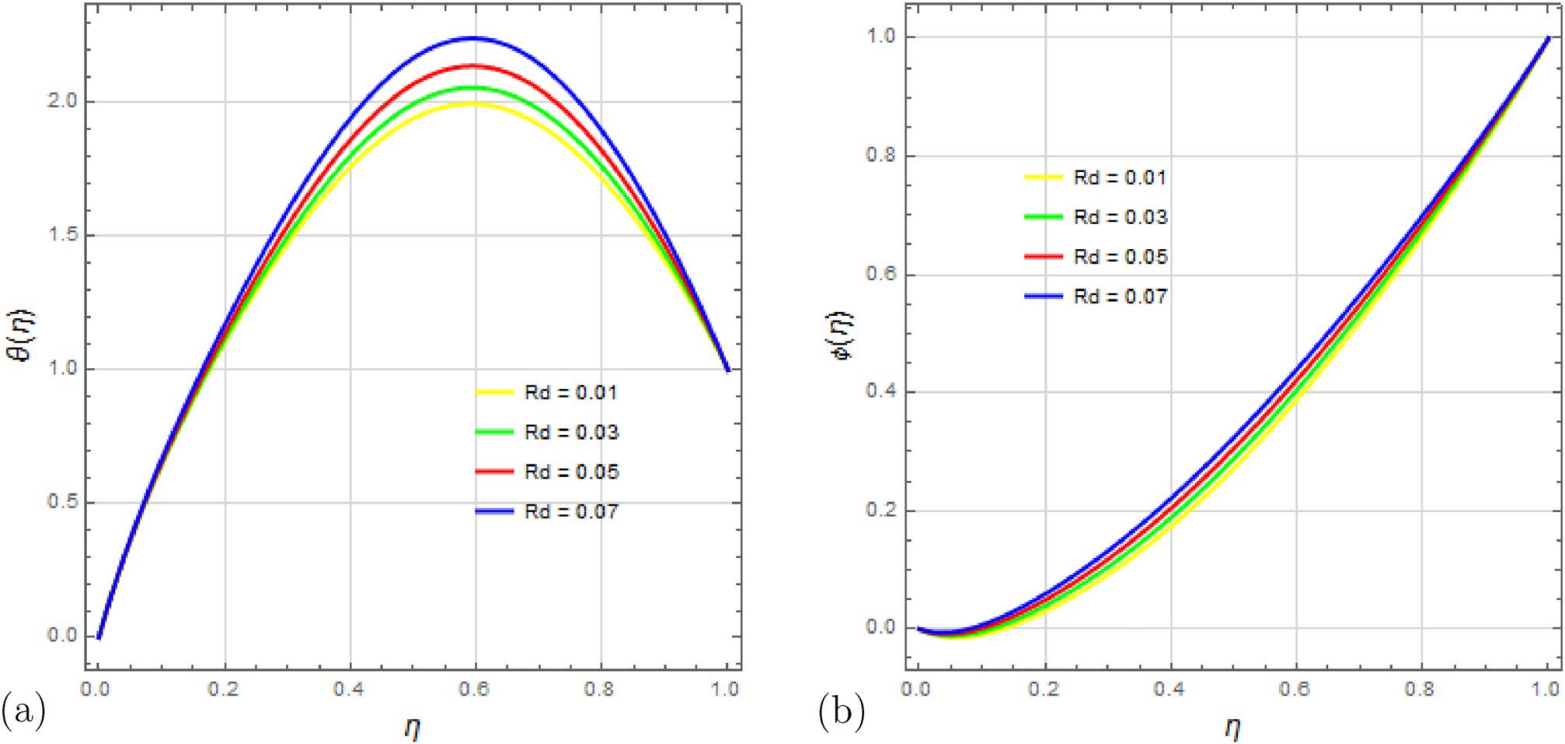
Impact of Radiation parameter $$ R_d $$ on temperature profile $$\theta (\eta )$$ and concentration profile $$\phi (\eta )$$ with $$\Omega =0.5$$, $$D_u=0.5$$, $$S_q=0.5$$, $$ S_c=0.5 $$, $$ S_r=0.5 $$, $$ S=1$$, $$ M=5 $$, $$P_r=0.5$$, $$E_c=0.5 $$ and $$\delta =0.1$$.

**Figure 15:**
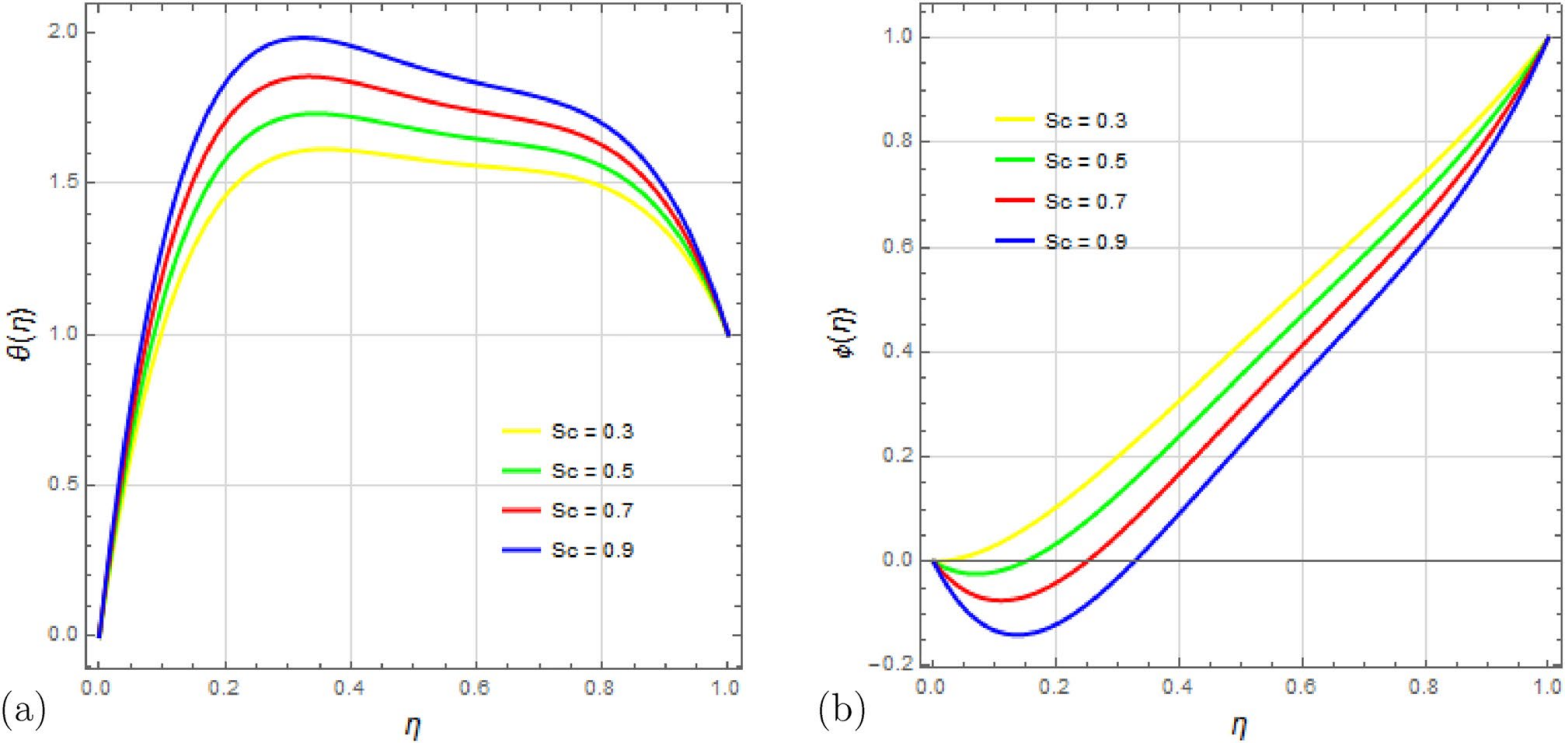
Impact of Schmidt number $$ S_c $$ on temperature profile $$\theta (\eta )$$ and concentration profile $$\phi (\eta )$$ with $$\Omega =1$$, $$D_u=1.5$$, $$S_q=0.5$$, $$ R_d=0.5 $$, $$ S_r=0.5 $$, $$ S=1$$, $$ M=0.5 $$, $$P_r=2.5$$, $$E_c=0.5 $$ and $$\delta =0.1$$.

The influence of the rotation parameter $\Omega$ on the velocity distribution and temperature distribution $\theta (\eta )$ is shown in Fig. 8a–d. In Fig. 8a, the velocity component $f(\eta )$ exhibits negligible variation with the rotation parameter $\Omega$ for $0<\eta <0.6$, while a slight decay is observed for $f(\eta )$ in the range $0.6<\eta <1$. Figure 8b illustrates that changing the rotational parameter $\Omega$ affects the velocity profile $f'(\eta )$ in three distinct regions: a slight increase in the lower half of the channel, a decrease in the center, and another increase in the upper half region. Figure 8c shows that altering the rotation parameter leads to a decrease in the velocity field $g(\eta )$, with a minimum value at the channel’s center. The impact of the rotation parameter $\Omega$ on the temperature profile and concentration profile is depicted in Figs. 8d and 9, respectively. Increasing the rotation parameter $\Omega$ results in a rapid increase in the temperature profile $\theta (\eta )$, while the concentration profile $\phi (\eta )$ shows a slower increase. Figures  10, 11, 12, 13, 14, 15 illustrate the effects of varying the Soret number $S_r$, Dufour number $D_u$, Prandtl number $P_r$, Eckert number $E_c$, radiation parameter $R_d$, and Schmidt number $S_c$ on the temperature profile $\theta (\eta )$ and concentration profile $\phi (\eta )$.

Figure 10a,b show the impact of the Soret number $S_r$ on the temperature and concentration distributions. $S_r$ represents the ratio of temperature difference to concentration and it is observed that it has a direct relationship with the temperature profile $\theta (\eta )$; increasing $S_r$ leads to a rise in temperature. Conversely, an inverse relationship is seen in the concentration profile $\phi (\eta )$; higher values of $S_r$ cause the concentration to decrease.

The variation of the Dufour number $D_u$ on temperature and concentration distributions is depicted in Fig. 11a,b. The Dufour effect represents the inverse phenomenon of the Soret effect, where changes in temperature are induced by concentration gradients. It is observed that an increase in the Dufour number $D_u$ intensifies the temperature gradient due to a higher rate of energy transfer, while the concentration gradient decreases. The rising Dufour number results in a reduction in the temperature difference between the fluid and the wall, leading to more heat being transferred to the fluid and affecting its viscosity, which in turn causes the concentration profile to decrease.

The behavior of the Prandtl number $P_r$ on temperature and concentration fields is shown in Fig. 12a,b. The Prandtl number represents the ratio of momentum diffusivity to thermal diffusivity. It is observed that with an increase in $P_r$, the temperature profile rises rapidly due to significant viscous dissipation effects, while the concentration profile $\phi (\eta )$ declines rapidly.

The impact of the Eckert number $E_c$ on the temperature $\theta (\eta )$ and concentration $\phi (\eta )$ is examined in Fig. 13a,b. The Eckert number relates the kinetic energy in the flow to enthalpy. As the Eckert number increases, the temperature rises due to internal particle friction converting mechanical energy into thermal energy. With a higher $E_c$, the concentration profile decreases.

Figure 14a,b illustrate how the radiation parameter $R_d$ affects the temperature $\theta (\eta )$ and concentration $\phi (\eta )$ fields while keeping the other parameters constant. As $R_d$ increases, the mean absorption coefficient decreases, resulting in more heat being transferred to the fluid. This leads to an increase in $\theta (\eta )$, but the fluid temperature for the stretched surface reaches a certain point and remains constant for a while. Additionally, the concentration rises at a slower rate compared to temperature.

Lastly, Fig. 15a,b depict the effects of the Schmidt number $S_c$ on temperature and concentration profiles. Schmidt’s number represents the ratio of kinematic viscosity to diffusion coefficient. It is evident that the fluid temperature increases as Schmidt numbers rise, while the concentration exhibits a reverse tendency. Higher Schmidt numbers lead to reduced concentration due to their inverse relationship with diffusion coefficient; a higher Schmidt number corresponds to a lower diffusion coefficient, resulting in a decrease in concentration.

## Conclusion

The effects of viscous dissipation and Joule heating on the 3-D MHD rotating squeezed flow of Newtonian fluid in a channel with a lower stretched permeable wall are investigated in this study. The impact of heat radiation is also maintained. The phenomenon is modelled with the help of coupled governing equations of continuity, Navier-Stokes, and heat and mass transfer. Further, the heat and mass transport properties are considered in the presence of the Soret and Dufour effects for 3-D squeezing MHD flow of an unsteady fluid. They are then converted into ordinary differential equations using the similarity transformation, and the Mathematica BVPh 2.0 package is used to solve them analytically via the homotopy analysis method. The influence of various parameters was shown using graphs and tables, and the error analysis was carried out up to the $10^{-40}th$ order. It has been concluded that

- The velocity components $f'(\eta )$, and $g(\eta )$ exhibit an increasing tendency as the squeezing parameter $S_q$ is raised.
- When the suction parameter *S* is increased, the velocity components $f'(\eta )$ along the x-axis decrease, while the velocity component $f(\eta )$ along the y-axis increases.
- As the magnetic number *M* rises, the velocity component $g(\eta )$ drop, whereas it is demonstrated that the velocity component $f'(\eta )$ falls as *M* rises but begins to rise as $\eta \rightarrow 1$.
- It is found that increases in the rotation parameter $\Omega$ have no impact on the velocity profile $f(\eta )$ for $0<\eta <0.6$, but $f(\eta )$ shows a slight decay for $0.6< \eta <1$. However, by increasing the rotational parameter $\Omega$, the velocity profile $f'(\eta$) exhibits a slight increase in the bottom half of the channel, a drop in the middle of the channel, and an increase in the top half area. Also, altering the rotation parameter $\Omega$ lowers the velocity field $g(\eta$), and the channel’s center can display the minimal value of $g(\eta )$.
- In the case of temperature distribution, it is shown that temperature transfer has a direct relationship with the dimensionless parameters suction parameter, magnetic number, rotation parameter, Soret number, Dufour number, Eckert number, Prandtl number, radiation parameter, and Schmidt number, but squeezing parameter has an inverse relationship.
- In addition, it is determined that mass transfer rises with increasing rotational and radiation parameters but exhibits a diminishing pattern with rising magnetic, squeezing, and suction parameters, as well as with increasing Dufour, Soret, Eckert, Prandtl, and Schmidt numbers.

## Data Availability

All data generated or analysed during this study are included in this published article.

## List of symbols

*V*: Fluid velocity
*h*(*t*): Distance between plates at $t=0$
$$\sigma $$: Electric conductivity
$\nu$: Fluid’s kinematic viscosity
$$S_z$$: Squeezing number
$$\theta $$: Dimensionless temperature
*D*: Diffusion coefficient
*T*: Fluid temperature
$R_{m}$: Magnetic Reynold number
$$P_r$$: Prandtl number
$$D_u$$: Dufour number
$$R_d$$: Radiation parameter
*B*: Magnetic field
$\mu$: Fluid’s dynamic viscosity
$\mu _2$: Permeability of free space
$$\rho $$: Fluid density
*P*: Fluid pressure
$$\phi $$: Dimensionless concentration
$$\kappa $$: Thermal conductivity
*C*: fluid concentration
*S*: Squeezing number
$$S_{c}$$: Schmidt number
$$S_{rt}$$: Soret number
$C_{p}$: Specific heat at constant pressure

## References

[1] Alfv$$\grave{e}$$n, H. Existence of electromagnetic-hydrodynamic waves. *Nature* **150**(3805), 405–406 (1942).

[2] Hamza EA (1991). The magnetohydrodynamic effects on a fluid film squeezed between two rotating surfaces. J. Phys. D Appl. Phys..

[3] Siddiqui AM, Irum S, Ansari AR (2008). Unsteady squeezing flow of a viscous MHD fluid between parallel plates, a solution using the homotopy perturbation method. Math. Model. Anal..

[4] Domairry, G. & Aziz, A. Approximate analysis of MHD squeeze flow between two parallel disks with suction or injection by homotopy perturbation method. *Math. Probl. Eng.* (2009).

[5] Mohyud-Din ST, Khan SI, Khan U, Ahmed N, Xiao-Jun Y (2018). Squeezing flow of MHD fluid between parallel disks. Int. J. Comput. Methods Eng. Sci. Mech..

[6] Joneidi AA, Domairry G, Babaelahi M (2010). Effect of mass transfer on a flow in the magnetohydrodynamic squeeze film between two parallel disks with one porous disk. Chem. Eng. Commun..

[7] Hayat T, Yousaf A, Mustafa M, Asghar S (2012). Influence of heat transfer in the squeezing flow between parallel disks. Chem. Eng. Commun..

[8] Takhar HS, Chamkha AJ, Nath G (2002). MHD flow over a moving plate in a rotating fluid with magnetic field, Hall currents and free stream velocity. Int. J. Eng. Sci..

[9] Kurosaka, M. The oscillatory boundary layer growth over the top and bottom plates of a rotating channel (1973).

[10] Crane, L. J. Flow past a stretching plate. Zeitschrift f$$\ddot{u}$$r angewandte Mathematik und Physik ZAMP, 21(4), 645–647 (1970).

[11] Wang CY (1984). The three-dimensional flow due to a stretching flat surface. Phys. Fluids.

[12] Pavlov KB (1974). Magnetohydrodynamic flow of an incompressible viscous fluid caused by deformation of a plane surface. Magnitnaya Gidrodinamika.

[13] Munawar S, Mehmood A, Ali A (2012). Three-dimensional squeezing flow in a rotating channel of lower stretching porous wall. Comput. Math. Appl..

[14] Hayat T, Sajjad R, Alsaedi A, Muhammad T, Ellahi R (2017). On squeezed flow of couple stress nanofluid between two parallel plates. Results Phys..

[15] Bachok N, Ishak A, Pop I (2013). Stagnation point flow toward a stretching/shrinking sheet with a convective surface boundary condition. J. Frankl. Inst..

[16] Hayat T, Haider F, Muhammad T, Alsaedi A (2018). Darcy–Forchheimer squeezed flow of carbon nanotubes with thermal radiation. J. Phys. Chem. Solids.

[17] Sheikholeslami M, Ganji DD, Javed MY, Ellahi R (2015). Effect of thermal radiation on magnetohydrodynamics nanofluid flow and heat transfer by means of two phase model. J. Magn. Magn. Mater..

[18] Hayat T, Shehzad SA, Ashraf MB, Alsaedi A (2013). Magnetohydrodynamic mixed convection flow of thixotropic fluid with thermophoresis and Joule heating. J. Thermophys. Heat Transf..

[19] Rashidi MM, Abelman S, Mehr NF (2013). Entropy generation in steady MHD flow due to a rotating porous disk in a nanofluid. Int. J. Heat Mass Transf..

[20] Fiza, M., Alsubie, A., Ullah, H., Hamadneh, N. N., Islam, S., & Khan, I. Three-dimensional rotating flow of MHD Jeffrey fluid flow between two parallel plates with impact of hall current. *Math. Probl. Eng.* (2021).

[21] Alam MK, Bibi K, Khan A, Noeiaghdam S (2021). Dufour and Soret effect on viscous fluid flow between squeezing plates under the influence of variable magnetic field. Mathematics.

[22] Hayat T, Shafiq A, Alsaedi A (2016). Hydromagnetic boundary layer flow of Williamson fluid in the presence of thermal radiation and Ohmic dissipation. Alex. Eng. J..

[23] Hosseinzadeh K, Mogharrebi AR, Asadi A, Sheikhshahrokhdehkordi M, Mousavisani S, Ganji DD (2022). Entropy generation analysis of mixture nanofluid (H2O/c2H6O2)$$-$$Fe3O4 flow between two stretching rotating disks under the effect of MHD and nonlinear thermal radiation. Int. J. Ambient Energy.

[24] Memon, M., Shaikh, A. A., Siddiqui, A. M., & Kumar, L. Analytical solution of slow squeeze flow of slightly viscoelastic fluid film between two circular disks using recursive approach. *Math. Probl. Eng.* (2022).

[25] Shamshuddin, M. D., Mishra, S. R., B$$\acute{e}$$g, O. A., & Kadir, A. Viscous dissipation and Joule heating effects in non-Fourier MHD squeezing flow, heat and mass transfer between Riga plates with thermal radiation: variational parameter method solutions. *Arab. J. Sci. Eng.* **44**(9), 8053–8066 (2019).

[26] Mahanthesh B, Gireesha BJ, Manjunatha S, Gorla RSR (2017). Effect of viscous dissipation and Joule heating on three-dimensional mixed convection flow of nano fluid over a non-linear stretching sheet in presence of solar radiation. J. Nanofluids.

[27] Parand, K., Ghaderi, A., Yousefi, H., & Delkhosh, M. Solving magneto-hydrodynamic squeezing flow between two parallel disks with suction or injection using three classes of polynomials. *Palest. J. Math.* **6** (2017).

[28] Hayat T, Jabeen S, Shafiq A, Alsaedi A (2016). Radiative squeezing flow of second grade fluid with convective boundary conditions. PLoS ONE.

[29] Ahmed N, Khan U, Mohyud-Din ST (2017). Influence of thermal radiation and viscous dissipation on squeezed flow of water between Riga plates saturated with carbon nanotubes. Colloids Surf. A.

[30] Nadeem S, Akram S (2010). Slip effects on the peristaltic flow of a Jeffrey fluid in an asymmetric channel under the effect of induced magnetic field. Int. J. Numer. Methods Fluids.

[31] Hayat T, Alsaedi A (2011). On thermal radiation and Joule heating effects in MHD flow of an Oldroyd-B fluid with thermophoresis. Arab. J. Sci. Eng..

[32] Hayat T, Ullah I, Muhammad T, Alsaedi A (2017). Radiative three-dimensional flow with Soret and Dufour effects. Int. J. Mech. Sci..

[33] Chen, H., Chen, J., Geng, Y., & Chen, K. Three$$-$$dimensional boundary layer flow over a rotating disk with power$$-$$law stretching in a nanofluid containing gyrotactic microorganisms. Heat Transf. Asian Res. **47**(3), 569–582 (2018).

[34] Muzara H, Shateyi S (2021). MHD laminar boundary layer flow of a jeffrey fluid past a vertical plate influenced by viscous dissipation and a heat source/sink. Mathematics.

[35] Shamim Z, Shahzad A, Naseem T (2022). Flow and heat transfer of power law fluid over horizontal stretching cylinder with partial slip condition and thermal radiation. Int. J. Emerg. Multidiscip. Math..

[36] Krishna, M. V., & Chamkha, A. J. Hall effects on MHD squeezing flow of a water-based nanofluid between two parallel disks. J. Porous Media **22**(2) (2019).

[37] Zhao, Y., &Liao, S. User Guide to BVPh 2.0. School of Naval Architecture, Ocean and Civil Engineering, Shanghai, 40. http://numericaltank.sjtu.edu.cn/BVPh.htm (2002).

[38] Asifa, Anwar, T. & Kumam, P. *et al.* Exact solutions via Prabhakar fractional approach to investigate heat transfer and flow features of hybrid nanofluid subject to shape and slip effects. *Sci. Rep.* **13**, 7810 (2023). 10.1038/s41598-023-34259-9. 10.1038/s41598-023-34259-9 PMC10183471 37183197

[39] Asifa, Anwar, T., Kumam, P. & Suttiarporn, P. *et. al.* A mathematical study on thermal performance of aluminum and titanium alloys based hybrid nanofluid using a multiparametric fractional operator. Case Stud. Therm. Eng. **45**(May), 102909 (2023).

[40] Anwar T, Kumam P, Muhammad S (2022). New fractional model to analyze impacts of Newtonian heating, shape factor and ramped flow function on MgO-SiO2-Kerosene oil hybrid nanofluid. Case Stud. Therm. Eng..

[41] Khan A, Shah RA, Alam MK, Ahmed H, Shahzad M, Rehman S, Ahmed S, Khan MS, Abdel-Aty AH, Zakarya M (2021). Computational investigation of an unsteady non-Newtonian and non-isothermal fluid between coaxial contracting channels: A PCM approach. Results Phys..

[42] Kamran Alam M (2021). Modeling and analysis of high shear viscoelastic ellis thin liquid film phenomena. J. Phys. Scr..

[43] Liao, S. Beyond Perturbation: Introduction to the Homotopy Analysis Method. Chapman and Hall/CRC (2003).

[44] Alam MK, Bibi K, Khan A, Fernandez-Gamiz U, Noeiaghdam S (2022). The effect of variable magnetic field on viscous fluid between 3-D rotatory vertical squeezing plates: a computational investigation. Energies.

[45] Waqas H, Farooq U, Ibrahim A, Kamran Alam M, Shah Z, Kumam P (2021). Numerical simulation for bioconvectional flow of burger nanofluid with effects of activation energy and exponential heat source/sink over an inclined wall under the swimming microorganisms. Sci. Rep..

[46] Khan A, Shah RA, Alam MK (2021). Flow dynamics of a time-dependent non-Newtonian and non-isothermal fluid between coaxial squeezing disks. Adv. Mech. Eng..

[47] Alam MK, Bibi K, Khan A, Noeiaghdam S (2021). Dufour and Soret effect on viscous fluid flow between squeezing plates under the influence of variable magnetic field. Mathematics.

[48] White FM (1991). Viscous fluid flow, mcgraw hill book company. N. Y..

